# Caring Cooperators and Powerful Punishers: Differential Effects of Induced Care and Power Motivation on Different Types of Economic Decision Making

**DOI:** 10.1038/s41598-017-11580-8

**Published:** 2017-09-11

**Authors:** G. Chierchia, F. H. Parianen Lesemann, D. Snower, M. Vogel, T. Singer

**Affiliations:** 1 Department of Social Neuroscience, Max Planck Institute for Human Cognitive and Brain Sciences, Leipzig, Germany; 2 Kiel Institute for the World Economy, Kiel, Germany; 3 Leipzig Research Center for Civilization Diseases, University of Leipzig, Leipzig, Germany

## Abstract

Standard economic theory postulates that decisions are driven by stable context-insensitive preferences, while motivation psychology suggests they are driven by distinct context-sensitive motives with distinct evolutionary goals and characteristic psycho-physiological and behavioral patterns. To link these fields and test how distinct motives could differentially predict different types of economic decisions, we experimentally induced participants with either a Care or a Power motive, before having them take part in a suite of classic game theoretical paradigms involving monetary exchange. We show that the Care induction alone raised scores on a latent factor of cooperation-related behaviors, relative to a control condition, while, relative to Care, Power raised scores on a punishment-related factor. These findings argue against context-insensitive stable preferences and theories of strong reciprocity and in favor of a motive-based approach to economic decision making: Care and Power motivation have a dissociable fingerprint in shaping either cooperative or punishment behaviors.

## Introduction

Standard economic theory assumes that decisions are driven by stable and context-independent preferences^1^. In contrast, motivation psychologists have long argued that human behavior is frequently driven by a number of fundamental motives, such as affiliation, power or achievement, which can be conceived both as dispositional traits and as context-sensitive psychological states, affecting the direction, intensity and persistence of behavior^2^. In fact, it has been shown that these motives can be contextually induced, even by situational features not directly related to the decision at hand, and that their influence can be measured through standard game theoretical paradigms involving financial social exchange^3^. However, little is known on how distinct motives may differentially affect economic decision making.

Here we aimed to assess whether distinct induced motives can impact different patterns of decision making, by comparing the behavioral impact of two classic psychological motives related to distinct goals: a motive related to Care (henceforth “Care”) and one related to power and status (“Power”). To assess the impact of Care and Power beyond idiosyncrasies of specific decision contexts, we used a factor analytic approach. Specifically, two recent studies^4, 5^ both revealed two similar “latent factors” of social economic decisions: one predicting cooperation in a number of domains^6^ - also referred to as a “domain-general” “cooperative phenotype”^4^ - and the other related to punishments of norm-violators. Here we thus aimed to assess whether distinct induced motives can impact latent factors of cooperation and punishment, rather than focusing on selected decision contexts.

Care refers to a motivation to non-instrumentally help others and related constructs have woven in and out of the psychological literature for decades, albeit sometimes with different connotations, such as “help/altruism”^2^, “nurturance”^7^, or “intimacy”^8^ – while Power has been defined as the motivation to be strong, to control and to influence others^9^. Care and Power have been found to differ at many levels of analysis. From an evolutionary perspective, Care could have emerged to support offspring care and then been deployed for promoting altruistic goals in general^10, 11^, while a Power motive could have especially been useful for competing over finite resources^12^. Moreover, while traditional economic theory assumes that both of these decisions emerge from stable and self-interested preferences, evolutionary models suggest how probabilistic cooperation and competition are likely to be adaptive^13, 14^, further raising the possibility that they are differentially mediated by distinct motives, such as Care and Power, associated with different contextual/external stimuli.

Care and Power have also been suggested to have a characteristic impact on a number of markers. For instance, at the perceptual level, they have been shown to affect vigilance for different facial expressions of high vs. low dominance^15^. At the emotional level, Care has been associated with feelings of warmth, compassion, affection, love, or friendliness^11, 16^, while Power has been associated with decreased compassion and increased social distance^3, 17^. At the cognitive level, Power has been associated with increased attentional focus^18^, decreased ability to take perspectives different from one’s own^19^, optimistically biased judgments^20^, and higher focus on gains than losses^21^; while Care has been associated with increased capacity to decode emotions from facial expressions^22^, and reduced focus on one’s own needs^23^. At the hormonal level, caring behaviors have frequently been associated with the oxytocin system^24^, while testosterone has been proposed to play a role in several Power or status-related behaviors^25^. Finally, on the level of behavior, Care motivation, empathic concern or compassion^16, 23^ have been associated with behaviors that altruistically benefit other individuals or groups^26, 27^ and with activation in affiliation and reward related neuronal networks^16^, while Power has frequently been associated to competitive or assertive behaviors^2^. In spite of this preliminary evidence, it remains unclear how Care and Power might systematically affect different factors of economic decision making as conceptualized and investigated in behavioral economics.

Notably, while some economic and evolutionary theories postulate that a single “behavioral propensity” or “tendency”, such as strong reciprocity^28, 29^, might underlie both cooperative behaviors *and* punishments of norm-violators, another stream of research on the contrary suggests that feelings of empathic concern or compassion towards one’s interaction partner can increase cooperation in social dilemmas, while at the same time decreasing punishments of norm violations in economic decisions^30–32^. Similarly, training in compassion has been linked to increased cooperative behavior^33^ and decreased likelihood to punish non-cooperators^34^.

As for Power, previous studies suggest that, in contrast to Care, it could *increase* punishment of norm-violations. In fact, punishing norm violators can result in reputational and status benefits and powerful punishers are also less likely to incur “counter-punishments”^35^. In line with this, rejections of unfair offers that are detrimental to all parties of a transaction (i.e., in ultimatum games) have been found to be predicted by self-reported assertiveness^36^ and induced power has been associated with more strict evaluation of moral transgressions^37^. Regarding cooperation, the differentiation between Care and Power is less clear-cut. In fact, Power-motivation could also increase economic cooperation in given circumstances, because subjects can increase their status by acting prosocially^38^. On the basis of this literature, we hypothesized that induced Power and Care should differentially affect behavior in economic decisions involving punishments but not necessarily those involving cooperation.

**Figure 1:**
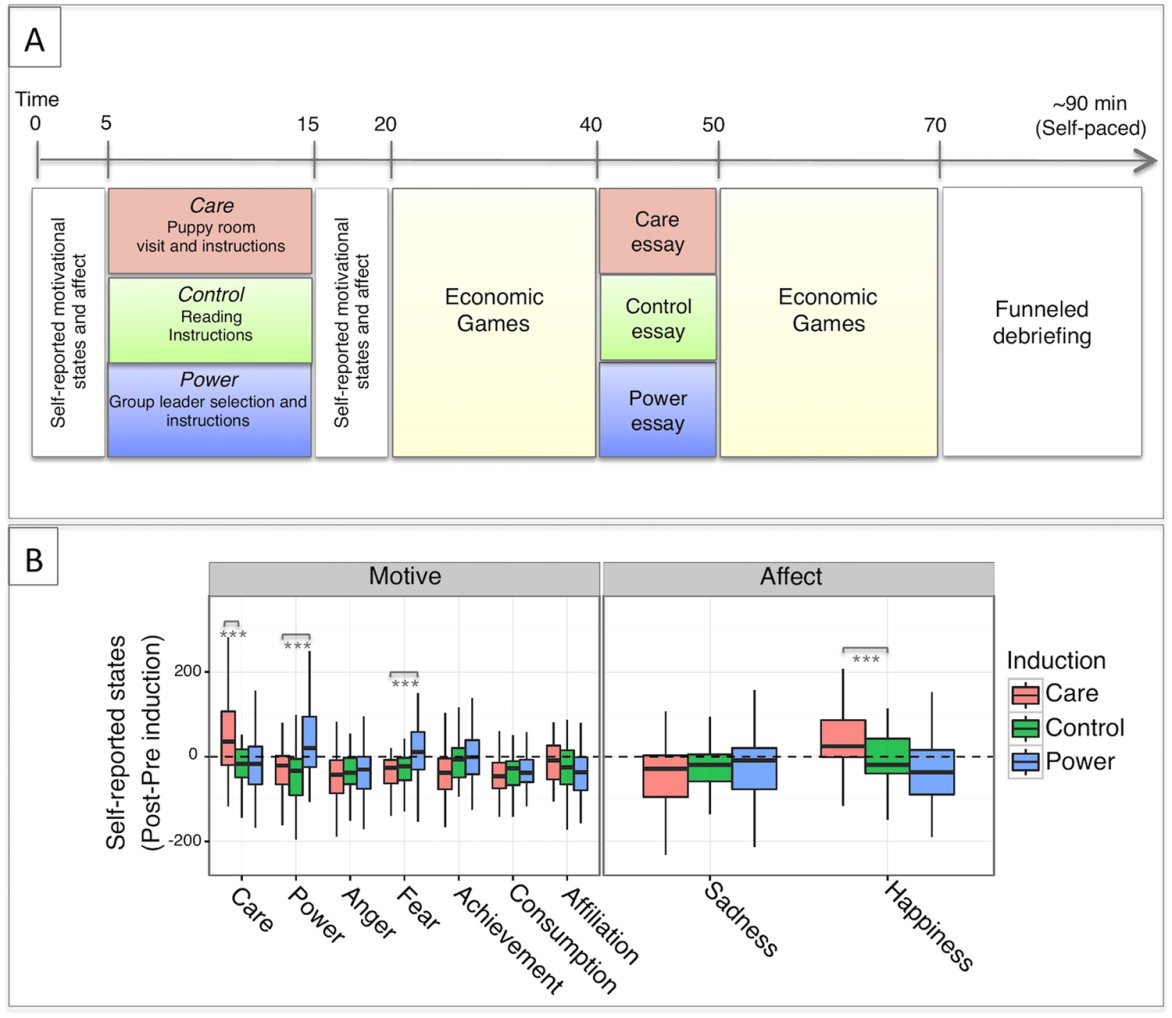
Panel A: Induction procedure. Panel B: Induction validation. After the care induction, participants reported higher feelings of care and happiness, relative to control. After the power induction, participants selectively reported higher feelings of power but also of fear, relative to controls. Visual analogue scales ranged from −350 to 350. ***p < 0.001.

To induce a Care motive we tested a novel induction method in which participants were informed about a therapy-dog training program requiring them to spend 10 minutes with a group of puppies (Fig. 1, panel A), with the alleged objective of getting the therapy dogs accustomed to interacting with different individuals. This induction was inspired from literature on the “Kindchenschema”, or “baby schema”: a set of infantile physical features that express vulnerability and that are known to trigger caretaking in observers^39^, even across different species^40^. Moreover, the psychosocial impact of human-animal interactions have been suggested to be mediated by oxytocin^41^. To sensitize participants to Power, we followed a frequently adopted paradigm (e.g., Lammers *et al*.)^3^ in which, on the basis of a bogus personality assessment, participants were selected as “leaders” of an upcoming group project. More specifically, participants were informed that leaders would evaluate the performance of their subordinates during a group project, and that this would determine their payoffs. Participants assigned to the control group were to read a passage of text. In each condition, participants also provided ratings on a set of motive and affect-related items (see Methods), once before knowing anything about these activities, and once after. The difference between pre- and post- induction ratings was used to validate the inductions. None of these announced activities would actually take place, and while participants prepared for them they were asked to take part in an allegedly separate experiment on economic decisions (see Methods).

## Results

### Validation of the motive inductions

A linear mixed effect model on change in motivational and affective ratings (i.e., difference scores between post-induction and pre-induction ratings) resulted in a highly significant interaction between the induction and the motive/affective state (F_(16,1536)_ = 4.59, p < 0.001), suggesting that the latter were differentially affected by the inductions (Fig. 1, panel B). Contrasts within the model revealed that, relative to Control, participants in the Care induction reported increased feelings of care (M = 86.42, 95% CI [52.47 120.37], p < 0.001) and happiness (M = 60.2, 95% CI [26.25 94.16], p < 0.001), while motivational state changes related to any of the other motive and mood states were not significantly affected by the induction (all p_s_ > 0.2). Similarly, relative to Control, participants in the Power induction reported an increase in feelings of power (M = 87.05, 95% CI [53.71 120.39], p < 0.001), as well as fear (M = 53.02, 95% CI [19.68 86.36], p < 0.001), while all other states were not significantly altered by the induction (all p_s_ > 0.14). The two inductions of Care and Power thus indeed succeeded in eliciting the target motives.

To investigate whether increases in feelings of power remained significant after controlling for increases in fear, we ran a new linear regression model estimating power change scores based on the fear change scores and the induction (Control vs. Power only). We found that the Power induction continued to explain increased feelings of power relative to Control, even while controlling for fear (F(1,130) = 21.97, p < 0.001). Likewise, we investigated whether increased care could be explained by increased happiness. To do so, we ran a new linear regression model estimating care change scores based on the happiness change scores and the induction (Control vs. Care only). We found that the Care induction continued to significantly explain the increased feelings of care (relative to Control), even after controlling for increases in happiness (F(1,135) = 11.13, p < 0.01). Moreover, the opposite was not true, namely, happiness no longer differed between the Care and Control inductions when controlling for increased care (F(1,135) = 11.13, p = 0.22).

### Two factors of economic behavior

**Figure 2:**
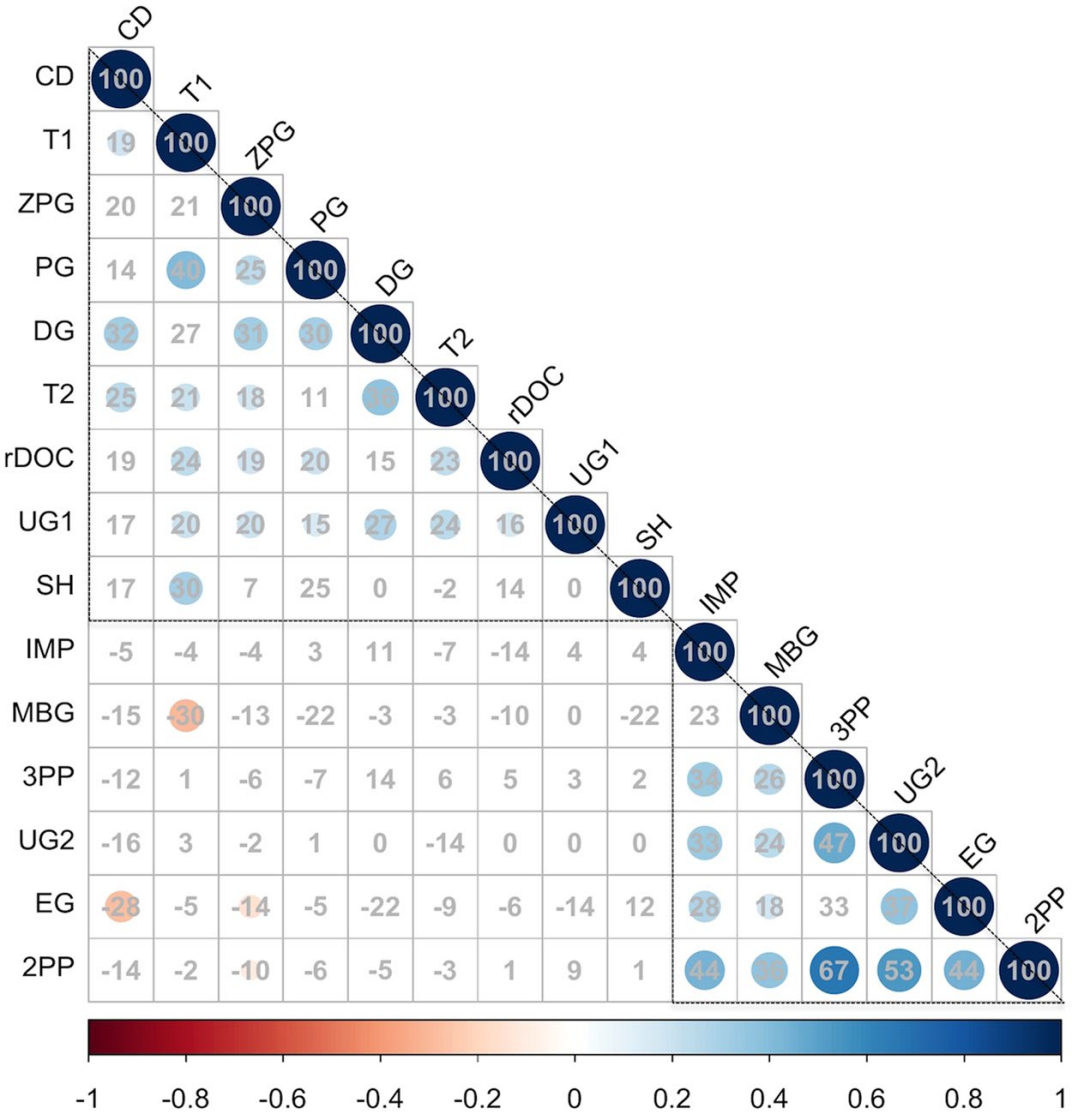
Correlation matrix of different economic decisions (i.e., “economic games”). Spearman’s coefficients (in percentage) of all possible correlations between economic games (n = 15). Colored cells represent significant correlations (at least p < 0.05, corrected for multiple comparisons with Holm’s method). Triangles are manually super-imposed to highlight two potential groups of economic decisions (ordered on the basis of their loadings on the correlation matrix’s first principal component). Game abbreviations: “T1” = amounts entrusted (as 1^st^ mover) in a trust game (i.e., “trust”); “T2” = average amount returned (as 2^nd^ mover) in a trust game (i.e., “trustworthiness”); “PG” = amount contributed in a public good game; “UG1” = amount offered (as 1^st^ mover) in an ultimatum game; “rDOC” = amount not taken from a common resource (in a dilemma of the commons); “DG” = amount given in a dictator game, “ZPG” = frequency of helping in the Zurich prosocial game; “CD” = average donation to charities, “SH” = frequency of cooperative decisions in a stag hunt; “2PP” & 3PP = average magnitude of punishments in 2^nd^ & 3^rd^ party punishment game; “UG2” =frequency of rejections as 2^nd^ mover in an ultimatum game; “EG” =frequency of entries in an entry game.

A (Spearman’s) correlation matrix (Fig. 2) suggested that two groups of economic behaviors were inter-correlated. Specifically, 1^st^ and 2^nd^ movers in the trust game - represented by average transfer rates (i.e., “trust” by 1^st^ movers) and average returns (i.e., “trustworthiness” by 2^nd^ movers) – average charitable donations, transfers in the dictator game, proposals as 1^st^ movers in the ultimatum game, average contributions to public goods, restraint in common resource dilemmas and helping in the Zurich prosocial game, all positively correlated. On the other hand, average punishment rates in the 2^nd^ and 3^rd^ party punishment games, frequency of rejections in the ultimatum and impunity games, entering in the entry game, all positively correlated with one another. Correlations of games *within* these two groups were all positive and, for the most part, significant, but correlations of games between the groups were for the most part absent or negative.

**Figure 3:**
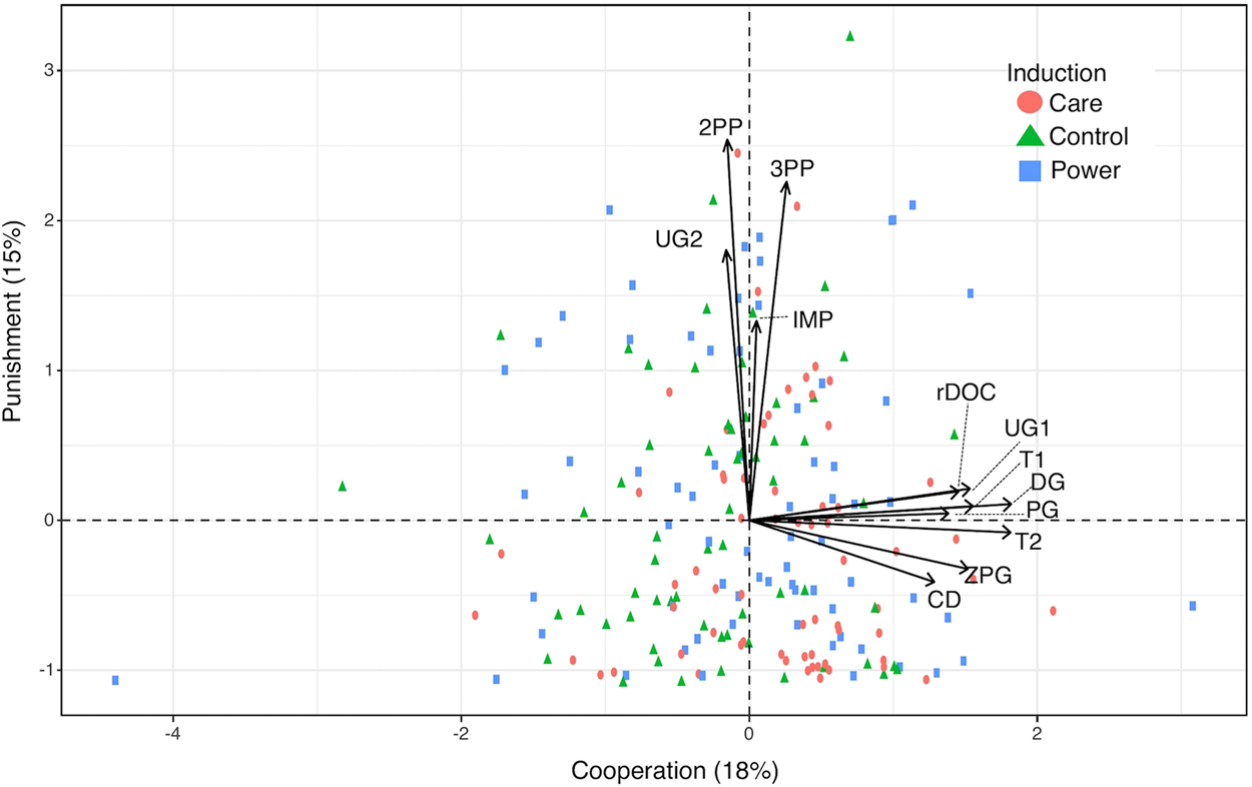
Factor analysis of behavior in economic games: two orthogonal factors (axes) recovered one third (33%) of the overall variance of the tested games (n = 12, represented by the arrows). This factor structure was also stable across the inductions. We label the 1^st^ factor (x-axis) “Cooperation” and the second (y-axis) “Punishment”. Individuals are colored based on the induction they took part in (red = “Care”, green = “Control”, blue = “Power”).

To test formally whether these groups of economic decisions could be related to distinct factors, we conducted a factor analysis (Fig. 3), following Peysakhovich *et al*.^4^ and Böckler *et al*.^5^. Three indexes (acceleration factor, very simple structure, and Velicer’s minimum average partial test) recommended retaining two factors, while three different indexes (optimal coordinates, parallel analysis and the eigenvalue criterion) recommended to retain three factors. However, since the third factor explained little additional variance (+5%), and since we were mainly interested in behavior on the first two factors as found by Peysakhovich and colleagues (2014), who only retained the first two factors, and Böckler and colleagues (2015), we retained the simpler two factor solution. As oblimin rotation left these first two factors virtually orthogonal (correlation of factors <0.04), we used the orthogonal Varimax rotation. The corresponding 2-factor solution revealed that each economic variable loaded clearly on at least one of the two factors (all loadings >0.3), with the exception of the stag hunt, which loaded poorly on both (both loadings <0.16). We thus excluded this variable and re-ran the analysis. As the resulting factor structure had satisfactory fit (RMSEA = 0.065 TLI = 0.85), we ran a confirmatory factor analysis to investigate whether this resulting factor structure was stable across the inductions/groups^42^. Measurement invariance protocols suggested that it wasn’t, due to the money burning game and the entry game, which loaded differently on each of the two factors, depending on the induction. We thus re-ran a third factor analysis excluding these variables. The resulting 2-factor structure continued to have satisfactory fit (RMSEA = 0.063 TLI = 0.88) and measurement invariance suggested that it was also stable across the inductions: neither the loadings, nor the intercepts significantly differed between them (both p_s_ > 0.1), thus allowing us to compare participants’ factor scores between the inductions. We took this as our final factor solution, in which 2 factors explained one-third (33%) of the variance in 12 economic variables.

This factor structure and loadings largely corroborated the groupings of economic variables individuated in the correlation matrix, as well as those individuated previously by Böckler and colleagues^5^ and Peysakhovich and colleauges^4^. Specifically, charitable donations, trust, trustworthiness, contributions in public goods, restraint in the dilemma of the commons, offers sizes in ultimatum games and helping in the Zurich prosocial game, all reliably loaded on factor 1 (all loadings >0.4), but not on factor 2 (all loadings in absolute value < 0.15) (see Table 1). Conversely, punishments in second and third party punishment games, as well as rejections in ultimatum and impunity games all loaded on factor 2 (all loadings >0.43), but not on factor 1 (all loadings in absolute value < 0.1). Following Peysakhovich and colleagues (2014), who describe the economic decisions loading on the first factor as “cooperative games”, we called factor 1, a “Cooperation” factor; while we called factor 2 a “Punishment factor” (also because the top loading variable on this factor was the 2^nd^ party punishment game). Another potential terminology could be the one used in Böckler *et al*.^5^, in which the first factor was described as capturing “altruistic” behaviors and the second related to the enforcement of “norms”.

**Table 1:** Exploratory factor analysis of 12 economic variables.

	F1: Cooperation	F2: Punishment	Com	Comp
Trust game (1^st^ mover)	**0.51**	0.03	0.26	1.01
Trust game (2^nd^ mover)	**0.60**	−0.03	0.36	1.00
Charitable donations	**0.42**	−0.14	0.20	1.20
Dictator game	**0.60**	0.04	0.36	1.01
Ultimatum game (1^st^ mover)	**0.50**	0.07	0.26	1.04
Public good game	**0.46**	0.02	0.21	1.00
Dilemma of the commons	**0.48**	0.06	0.23	1.04
Zurich prosocial game	**0.50**	−0.11	0.26	1.09
2^nd^ party punishment	−0.05	**0.84**	0.70	1.01
3^rd^ party punishment	0.09	**0.74**	0.56	1.03
Ultimatum game (2^nd^ mover)	−0.05	**0.59**	0.36	1.02
Impunity game	0.02	**0.44**	0.19	1.00
Proportion variance explained	0.18	0.15		

Standardized loadings (pattern matrix) based upon correlation matrix, communality (“Com”) and complexity (“Comp”) of each variable. Two factors, labeled “Cooperation” and “Punishment”, captured 33% of the total variance.

### The impact of induced Care and Power motives on economic behavior

**Figure 4:**
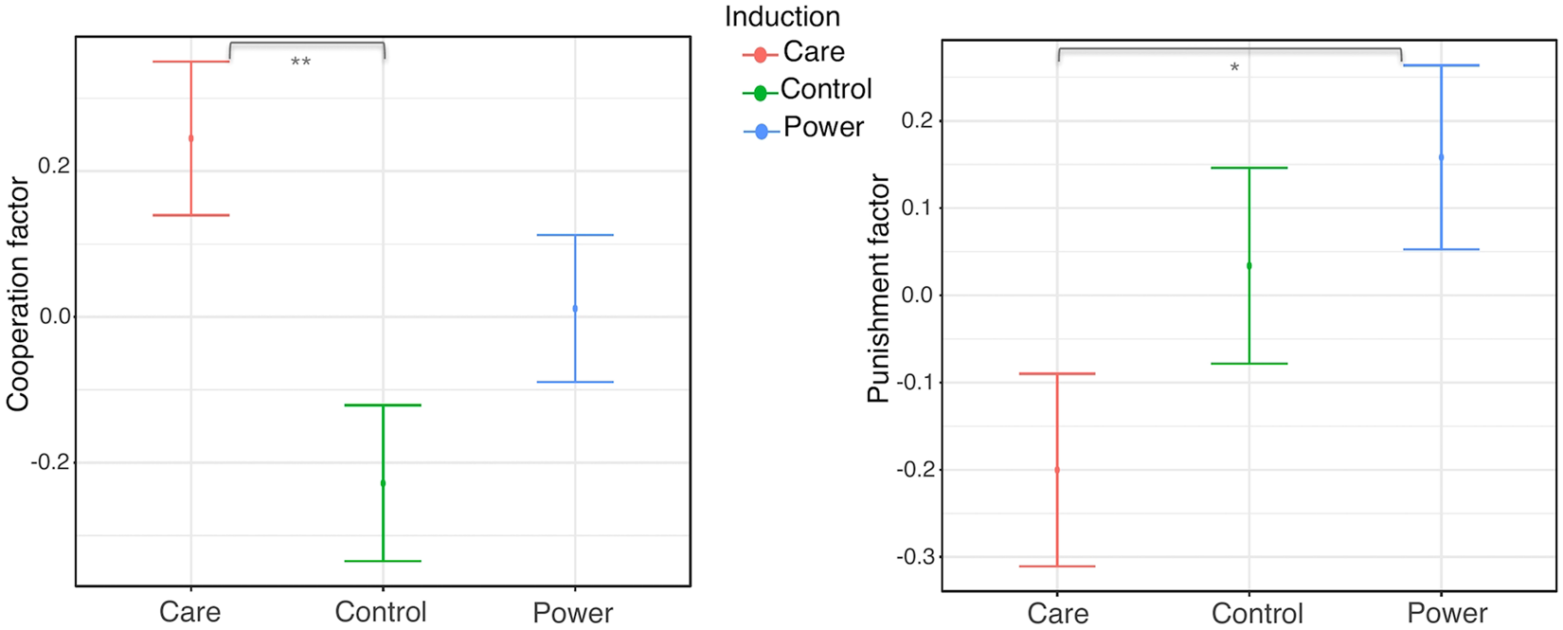
Model estimates predicting latent measures of cooperation and punishment in economic decisions as a function of induced Care or Power motives (vs. Control induction). Induced Care resulted in increased cooperation, relative to Control (left panel). Power and Care differentially affected punishment behaviors (right panel). Error bars represent 95% confidence intervals. **p < 0.01, *p < 0.05.

The factor analysis also enabled to obtain one pair of scores for each participant, which can also be thought of as a pair of coordinates, determining their position on a “cooperation × punishment space” (Fig. 4). To investigate the impact of the Care and Power inductions on subjects’ cooperative and punishing behaviors (subjects’ position on the factor space), we ran two models: one on the cooperation scores and one on the punishment scores. Both models revealed a significant main effect of the induction (Cooperation: F_(2,190)_ = 4.96, η_p_^2^ = 0.049, p < 0.01; Punishment: F_(2,190)_ = 4.56, η_p_^2^ = 0.045, p < 0.05) (Fig. 4). Contrasts within the cooperation model revealed that the significant main effect of the induction was due to induced Care significantly increasing cooperation rates relative to Control (M = 0.47, 95% CI [0.12 0.83]), p < 0.01), but not relative to induced Power (M = 0.24, 95% CI [−0.10 0.58], p = 0.23). Cooperative behavior after the Power induction also did not significantly differ from Control (M = 0.24, 95% CI [−0.11 0.58], p = 0.24). As for the punishment model, neither Care nor Power significantly differed from Control (Care: M = −0.23, 95% CI [−0.60 0.13], p = 0.29; Power: M = 0.13, 95% CI [−0.49 0.23], p = 0.67), however they significantly differed from one another, that is, the Power induction resulted in higher punishments than the Care induction (M = 0.36, 95% CI [−0.73 0], p < 0.05). In synthesis, induced Care resulted in increased cooperative behavior, while Power and Care differentially affected punishing in economic decisions.

Since the interaction between the induction and gender was not far from significance in the punishment model (F_(2,190)_ = 2.07, η_p_^2^ = 0.021, p = 0.13), we explored the interaction further with contrasts and found that punishments were clearly higher in Power than Care only in males (M = 0.66, 95% CI [0.14 1.19], p < 0.01), not in females (M = 0.06, 95% CI [0.43 0.56], p = 0.96). As this analysis was not hypothesized, and the interaction between gender and the induction was not significant, we analyze and discuss it further only in the supplementary material (Supplementary Material S6).

To investigate whether the inductions affected non-social decisions as well the decisions of interest above, we ran three further models predicting, respectively, risk attitudes, loss-aversion and temporal discounting, based on the inductions and gender. None of these non-social decisions were significantly affected by the inductions (all p_s_ > 0.13). We also re-ran the cooperation and punishment models above, adding these three non-social decision measures as covariates. Each of these models confirmed a significant main effect of the inductions (both p_s_ < 0.05). As a further control, we also re-ran the cooperation and punishment models whilst controlling for post-induction ratings of fear and happiness (as these differed between the inductions). The main effects of the inductions remained significant (both p_s_ < 0.05). Finally, these main results remained significant when excluding participants that, through post-experimental questionnaires, expressed some insight into the objective of the study (see Methods, and Supplementary Material S3, for details).

## Discussion

To bridge the fields of motivation psychology and behavioral economics and work towards a motive-based model of economic decision-making, we investigated how inducing two distinct motives, Care and Power, could differentially affect two types (or factors) of economic behaviors: cooperative versus punishment-based economic decisions, both having been linked in theories of “strong reciprocity”^28, 29^. We show that a novel Care induction, realized through the anticipation of a later interaction with a group of puppies, can increase a latent measure of cooperation based on a variety of cooperative game theoretical paradigms (e.g., as assessed by amounts transferred or returned in trust games, dictator games, public goods, charitable donations and others). Importantly, the induction of Care did not simultaneously increase a distinct and uncorrelated latent factor related to punishment of norm violators (as assessed by punishment frequency or magnitude in ultimatum games, 2^nd^ and 3^rd^ party punishing games and the impunity game). Instead, Power and Care differentially affected punishment-related behaviors, with these being more likely under induced Power than under induced Care. Thus, in the case of induced Care, a motive suggested to have evolved to assure survival of the off-spring^10, 11^, cooperative behaviors are more likely to occur (relative to controls). In contrast to this, in the case of Power, a motive suggested to have evolved to gain or maintain status/rank-related advantages^9^, punishing/norm-enforcing behaviors are more likely to occur, relative to Care. These findings argue against the assumption in standard neoclassical economic theory that decisions are driven by stable context-independent preferences^1^ and rather speak in favor of a motive-based model of economic decision making^14, 43^, in which different motives can be activated through different contexts, and once aroused, these motives can facilitate different action tendencies, some being more related to cooperation, others to punishment.

Our findings also inform previous behavioral economic and evolutionary theories positing that a same tendency, such as strong reciprocity, could explain engagement in both cooperative and “altruistic punishing” behaviors, as both behaviors can promote cooperation^28, 29^. Previous studies^5, 13, 36, 44^, however, had already argued that “punishment and cooperation may be separate phenomena” (Peysakhovich *et al*., 2014, p. 2)^4^, though these conclusions were based upon the observation that cooperation and punishment are frequently uncorrelated behaviors, while none attempted to manipulate these behaviors experimentally or linked them systematically to psychological motives. We thus add to this literature by showing that these two different classes of behaviors can be differentially manipulated through activation of different motives: an induced Care motive can increase cooperation without simultaneously increasing punishments, while punishments are more frequent in participants primed with Power than Care motivation. This result warrants caution when interpreting “altruistic punishments” as a sign of a *single* underlying preference such as in theories of strong reciprocity^28, 29^. Rather, our results suggest that they are more likely to stem from two different underlying motivational systems.

Plausibly, punishments of norm-violators need not stem only from a Power motive, but could also, for instance, emerge from motives not directly manipulated in this study, such as anger^45^. Likewise, cooperative behaviors need not stem only from inducing Care. In fact, as discussed in the introduction, it has been frequently suggested that Power-motivation too can foster cooperative behavior, especially if these cooperative behaviors can provide reputational/status benefits (e.g., Hardy and Vugt 2006)^38^. Our results however provide only partial support of this. In fact, we find that Care alone, and not Power, increased cooperation relative to control, though we also find that levels of cooperation did not significantly differ between the Care and Power conditions. We speculate that this might reflect a theoretical distinction between Care and Power-motivated cooperation, namely, that altruistic behaviors, intended to benefit others, are the final goal of Care motivation^30^, but are only instrumental for Power, which is ultimately related to increasing one’s own status and reputation and to controlling or influencing others. Thus, a novel implication of our results is that, if one aims to differentiate Care from Power-motivated decision making, one should either use paradigms clearly distinguishing between “pure altruism”^23^ from status or image scoring-based cooperation^38, 46^ or rather focus on punishment-related behaviors (e.g., by observing how subjects respond to norm-violations).

To conclude, we show that we could successfully induce two distinct psychological motives, Power and Care, and that activation of these motives differentially affected two different classes of economic decisions, one latent factor related to cooperative decisions, and another factor related to punishing norm violators in game theoretical paradigms. This pattern of findings challenges both traditional economic assumption of stable, context-insensitive preferences underlying economic decisions, as well as theories of strong reciprocity which suggest a same tendency drives both cooperation as well as the punishment of free-riders. These findings rather inspire the formulation of new motivation-based economic decision making models making formalized predictions about the forms of decisions that will arise, given a certain context or institutional setting and the activation of specific motives and action tendencies. Our research thus emphasizes the importance of contexts and motives in decision making, pointing to the potential of society, institutions and companies to shape different types of social behaviors in fostering, for example, either Care and resulting altruism, or Power motivation favoring norm-reinforcement and punishment.

## Methods

### Participants

198 participants (mean age = 27.1, SD = 4.8, 96 males) were randomly assigned to one of three groups: a “Care” group (N = 65,3 mean age = 26.8, SD = 4.2, 31 males), a “Power” group (N = 70, mean age = 26.6, SD = 4.3, 34 males), and a “Control” group (N = 63, mean age = 27.8, SD = 5.7, 31 males). Gender distribution or age did not significantly differ between any of the three groups (all p_s_ > 0.17). Participants were recruited via email through the Max Planck’s participant database. Due to a programming error, the decisions of two participants for one economic environment (i.e., the 2^nd^ party punishment game) were not recorded. No participants were excluded from the analysis. A separate group of 127 participants (mean age = 29.4, SD = 6.2, 61 males) was recruited to pilot and validate the motivational inductions, that is, to assess whether they succeeded in enhancing subjective feelings related to the intended motives. However, as those results were highly comparable to the ones in this paper they will not be discussed further. The datasets generated during and/or analysed during the current study are available from the corresponding author on reasonable request. All participants provided informed consent for the treatment and publication of their anonymized data. All assessments were approved by the Research Ethics Committee (agreement number 090-15-09032015) of the University of Leipzig, Germany. All experiments were performed in accordance with relevant guidelines and regulations.

### Motive inductions

The motive inductions were matched in terms of the sequence of events and duration. Specifically, as soon as participants entered the lab (thus before knowing anything about the upcoming activities) they provided base line ratings (on a visual analogue scale ranging −350 to 350) on the motivational states of interest (i.e., Power and Care-related states), as well as a number of control motives (i.e., Achievement, Affiliation, Anger, Fear, Consumption) and affective states (i.e., happiness and sadness). The Care-related items were “caring”, “protective”, “kind-hearted”, “cordial”, “helpful”, “affectionate”, “sympathetic”, and “consoling”; the while power-related words were “mighty”, “dominant”, “authoritarian”, “firm”, “influential”, “condescending” and “officious” (for details on the other items see Supplementary Material S5). After providing these baseline ratings, the induction phase began: participants in the Power induction completed a bogus questionnaire while participants in the Care induction saw a video that depicted puppies interacting with alleged participants (actually the puppy owners) in the Max Planck Institute during one of our previous experimental sessions. In both inductions, participants were then brought separately into a different room for further instructions. In the Care induction, they were introduced to the facilities (e.g. they visited the same “puppy room” they had seen in the video etc.) and were told that the puppies were to interact with them only once and would have thus only been brought in later, during the actual test session. To parallel this, in the Power induction, participants were brought to a distinct room, where an alleged psychologist gave them feedback on their previously taken personality assessment. In reality, each participant was nominated “director” of the upcoming group project. Finally in the Control induction, participants were brought to a room where they tried out the microphone for the upcoming recording. After being informed of these respective activities, participants came back to the computer lab and provided new ratings on the same motive-related and affective items seen previously. After providing these test ratings, participants in each of the three inductions were asked to take part in a different experiment on economic decision making, while they waited for the announced activities to take place.

### Awareness questionnaire

To control for potential experimental demand effects, at the end of the experimental session, we had participants take part in an “awareness questionnaire”, in which participants were given progressively clearer hints about the nature of the experiment (see Supplementary Material S3, for details). For instance, an early question asked participants what they thought the objective of the experiment was, while a subsequent one more specifically asked whether they believed any part of the experiment might have influenced another, and a subsequent one still asked them if they ever had the feeling of being deceived during the experiment. We had participants privately provide open written answers and then had two independent experimenters review them. We then re-ran our main analysis of interest using two exclusion criteria. A first excluded participants that expressed relevant understanding of how the announced activities were intended to affect their economic decisions (e.g., “The puppies made me nicer”), while a second more stringent criteria excluded participants that, when explicitly asked, admitted that the activities could have affected their decisions, but not in a way that was pertinent to our experimental hypotheses (e.g., “the puppies made me happy”). Our results were unchanged by either of these exclusions (see Supplementary Material S3, for details).

### Game theoretical paradigms

Following Peysakhovich *et al*.^4^ and Böckler *et al*.’s^5^, which identified a “cooperative” or “altruistic” factor, respectively, we included a dictator game (in which participants decided how much money, if any, to transfer to a passive recipient), a trust game (in which, as 1^st^ movers, participants decided how much money, if any, to entrust to a second mover, and as 2^nd^ movers decided how much, if anything, to give back to the 1^st^ movers), charitable donations (in which participants decided how much to donate to various charities) and the “Zurich prosocial game” (in which participants decided how much to help their counterparts in a virtual maze). In addition to these, inspired by the literature on cooperation, we had participants take part in a public good game (in which they decided how much money to contribute to a public good), a common resource dilemma (in which they decided how much to take from a common resource) and a stag hunt (where they chose between a potentially high paying but uncertain option, that required coordinating with others, or a lower paying but safe alternative that did not require coordination) (details of all game-theoretic paradigms are available in the Supplementary Material S2). As for the punishment-related games, we again followed the same two studies^4, 5^, and adopted the 2^nd^ and 3^rd^ party punishment game (in which participants observed how much another player transferred to themselves or a third party – 2^nd^ and 3^rd^ mover variants, respectively –and, on the basis of this, decided how much money, if any, to invest to “punish” them, that is, to decrease their payoff) and an ultimatum game (in which, as 1^st^ movers/proposers, participants decided what proportion of a monetary prize they would offer to a second mover and, as second movers/recipients, they decided whether to accept or reject proposals made by the proposers: if they accepted the monetary prize was split as proposed, if they rejected, both players received nothing). In addition to these, to investigate behaviors that have been related to competition or status, but are not confined strictly to punishments, we added an impunity game (which was identical to the ultimatum game with the exception that participants only played as second movers and if they rejected, the 1^st^ mover still kept what he/she proposed, while participants earned nothing), an entry game (which was identical to the stag hunt but was based on anti-coordination, thus if both participants chose the uncertain option, both earned nothing), and a money burning game (in which participants decided how much to spend to decrease the payoff of others). Finally, to control for the potential impact of our inductions on attitudes that regard non-social decisions (i.e., decisions that do not involve others), we measured risk attitudes, loss aversion and temporal discounting (see Supplementary Material S2 for details). Exploratory variants of the charitable donations game and the dictator game were piloted but not analyzed, as they are relevant to a different study. The economic games were presented in pseudo-randomized order (see Supplementary Material S4 for details) and participants were informed that they would be paid for one randomly determined decision, at the end of the experiment. Instructions of the game-theoretic paradigms are available in the supplementary material (Supplementary Material S7).

### Statistical analysis

Before analyzing the impact of the inductions on economic decisions, we aimed to validate the motive inductions by comparing motivational and affective ratings before and after the inductions. To do so, we, first, subtracted pre-induction ratings from post-induction ratings, in order to obtain a “change score” for each item. Then, we averaged over the items related to the same motivational/affective state, thus obtaining 9 rating change scores per participant. These difference scores were then compared between the inductions with a hierarchical mixed effect model that predicted change scores based on the following fixed effect terms: gender, the induction (with levels: “Care”, “Control”, “Power”), the motivational/affective state (9 levels) and their interaction - as well as participants as random intercepts.

After validating the motivational inductions we analyzed their impact on economic decisions. To do so, we first obtained a single variable for each game-theoretical paradigm (e.g., averaging over multiple trials when possible, see Supplementary Material S2 for details). Second, after determining the optimal number of factors to retain by comparing a number of standard indexes (eigenvalue criterion, parallel analysis, optimal coordinates, acceleration factor, very simple structure and Velicer’s minimal average partial test), we submitted these variables to factor analysis (for which a 10:1 subject-to-ratio is recommended - e.g., Velicer & Fava, 1998, p. 232 -, whereas our design involved a higher ratio of 13.2:1, suggesting an adequate sample size)^47^. Third, before comparing factor means between groups (Hirschfeld & Brachel 2014, p. 1^42^, argue that “if measurement invariance has been established for a measure, observed mean differences can be attributed to differences in underlying constructs between the groups”), we investigated whether the obtained factors structure was stable across the inductions/groups via confirmatory factor analysis, if not, we aimed to individuate and remove those economic variables that made the structure unstable^42^. We report standard fit and reliability indices of the final factor solution (i.e., the “root mean square error of approximation, or “RMSEA”, and the Tucker-Lewis index, or “TLI”). As a final step, we extracted participants’ scores on each of the obtained factors, and investigated whether these differed between the inductions with univariate linear regression models. We ran one model for each attested factor. These models predicted participants’ scores on the basis of the following terms: the induction, gender and their interaction. We then probed significant omnibus tests (as assessed by type III Anova) with contrasts, of which we report 95% confidence intervals and p-values (corrected with the Tukey method). Factor analysis was performed with the “fa” function (in the “Psych” package)^48^, while the stability of the factor structure across inductions/groups was assessed through confirmatory factor analyses, to which we applied the “measurementInvariance” function (in the “semTools” package)^42^. All analyses were carried out in R (R team). Stimuli were prepared and administered in Presentation (Neurobehavioral Systems, Inc.).

## Electronic supplementary material

Supplementary material
