# Supplementary material for: Caring Cooperators and Powerful Punishers: Differential Effects of Induced Care and Power Motivation on Different Types of Economic Decision Making
Source: Sci Rep. 2017 Sep 11;7:11068. doi: 10.1038/s41598-017-11580-8 (PMC5594000; doi:10.1038/s41598-017-11580-8)
Supplement: Supplementary file 1 — Supplementary material [file 41598_2017_11580_MOESM1_ESM.pdf]

Caring Cooperators and Powerful Punishers: Differential Effects of Induced Care and Power Motivation on Different Types of Economic Decision Making

Chierchia G.[a*], Parianen Lesemann F.[a], Snower D.[b], Vogel M.[c], Singer T.[a]

a. Department of Social Neuroscience, Max Planck Institute for Human Cognitive and Brain Sciences, Leipzig, Germany;

b. Kiel Institute for the World Economy, Kiel, Germany;

c. Leipzig Research Center for Civilization Diseases, University of Leipzig.

* Corresponding author

Supplementary material

S1 Detailed description of the experimental inductions

Care. Participants were informed that the institute was collaborating with a clinical institute that made use of therapy-dogs in their sessions. To be trained, the therapy dogs, as puppies, had to become accustomed to spending time with different people. Participants were thus asked to take turns for 10 minutes with 4 puppies in a "puppy room". Instructions were provided through a video which was shot in the adjacent "puppy room" and depicted a number of (alleged) participants (actually the puppy owners) interacting with the puppies. After receiving these instructions, one by one, the participants were brought to the puppy room, which contained a wooden fence and dog toys scattered on the ground. They were told that the puppies would only be brought in later for the actual training session and given some further instructions on the upcoming session (e.g., they were shown where the puppy snacks were).

After being brought back to the computer lab, participants were informed that, while the puppies were being brought into the room, they would take part in a second study on economic decision making, in which they could earn an additional payment, depending on the outcome of their decisions. While the experimenters set up the economic decision making experiment, they minimized a window that had previously occupied the entire screen. In doing so, they revealed the desktop, which was intended to act as a reminder of the therapy dog program and depicted a sleeping puppy held in a hand. While participants made their decisions, this desktop was not visible as the decision screen covered it. However, as soon as participants confirmed their choices, the decision screen shrunk to a smaller window that stated "the response is being processed" and depicted a progress bar (that filled up in 2 seconds). This smaller window made it possible to casually re-present the desktops in the background. Finally, half-way through the suite of economic games, participants answered a 10 minute questionnaire asking them to describe any previous experience they had with puppies. It also asked them whether they knew anyone with disabilities that might profit from dog-therapy and how the puppies might help these people.

Power. The power induction was inspired from Lammers and colleagues (2011, experiment 1). After taking part in a (bogus) personality assessment, one by one, participants were then

brought into another room, where an alleged professional psychologist told them that the assessment was a “leadership test” to select the leaders of an upcoming group project. Leaders were to supervise and evaluate the work of the others, and this evaluation would contribute to determining their payoff at the end of the group project. As for the Care induction, after returning to the computer lab, a power-related desktop was revealed. This desktop depicted a man in a suit and briefcase looking out of the window from a skyscraper and used the German word “stark” (“powerful”) as an acronym of the upcoming group project. As for the Care induction, this desktop reappeared after participants confirmed their choices. As for the care induction, half-way through the economic games, to prepare for the upcoming group project, participants answered a 10 minute questionnaire asking them to describe any previous experience they might have had where they had an influence over other people.

Control. Participants were told that we were recording material for a children’s education program, in which different individuals read non-challenging excerpts of the child text-book. As for the care and power inductions, one by one, participants were brought into the recording room to introduce them to their task. The desktops used did not depict anything. As for the care and power inductions, half-way through the economic games, participants spent 10 minutes describing a set of nature-related images that had been proposed to be accompany the text they were to read.

S2 Description of the game theoretical parameters

	**Game theoretical paradigms**	**Description**	**Parameters (n=number of trials)**
1	Dictator game (“DG”) (Engel, 2010 for a meta-analysis)	Subjects are endowed with a euro amount and decide how much, if any, of their endowment to transfer to an anonymous counterpart.	Endowment=€10.00<br><br>(n=1)

2	Stag hunt ("SH") (Camerer, 2003, chapter 7 for a review)	Randomly paired participants are to choose between (the same) two options: a safe but low paying option and a potentially higher paying but uncertain option. If both choose the high paying option, both earn the high payoff. However, if one chooses the high paying option and the other does not, the one that chose the high paying option receives nothing at all, while the low paying option results in a secure payoff (i.e., regardless the choice of one's counterpart). Participants made several decisions but never received feedback on their outcome. The value of the high paying option was kept fixed, while the value of the low paying option varied for each decision.	Low paying option value varied between 0 and 15€, in steps of 1€<br><br>High paying option value was kept fixed and was always worth 15.00€ o 0, depending on the choice of one's counterpart.<br><br>(n=16, randomized order)
3	Public good game (Zelmer, 2003 for a meta-analysis)	Groups of three participants decide how much of an endowment to contribute, if anything, to a "pubic good". Contributions are summed and multiplied by a given factor and the resulting amount is split equally among players, regardless of how much they contributed.	Endowment=€30.00<br>Multiplier factor = {2,3}<br><br>(n=2, no randomization)
		As first movers (the "proposer"), participants decide how much of an	Endowment=€10.00

4	Ultimatum game (as 1st mover, “UG1”, and 2nd mover, “UG2”) (Camerer, 2003, for a review)	endowment, if anything, to propose to a second mover.	(n=1)
5		As second movers (“recipients”), participants observe the proposal and decide whether to “accept” or “reject”. If accepted, each player receives the endowment share proposed by the proposer. If rejected, both players earn nothing.	Observed proposals = {0.2, 1.1, 1.7, 3.1, 4.1, 4.9}<br><br>(n=6, randomized order)
6	Third party punishment (“3PP”) (Fehr & Fischbacher, 2004)	A first mover decides how much of an endowment to transfer to a randomly matched counterpart.<br><br>Participants play the role of a 3rd unaffected party, who observe how much is transferred and decide how much of an endowment to spend, if anything, to punish the 1st mover.<br><br>This amount is multiplied a given factor and subtracted from the 1st mover’s payoff. Participants only played the 2PP as the 3rd unaffected party.	1st mover endowment=€15.00<br><br>2nd mover endowment=€5.00<br><br>Observed transfers = {0.2, 1.6, 2.5, 3.6, 6.1, 7.4}<br><br>Multiplier factor=3<br><br>(n=6, randomized order)
7	Trust game (as 1st	A 1st mover (“truster”) decides how much of an endowment, if anything, to transfer to a 2nd mover (“trustee”).	Endowment=€10.00<br>Mulitpier factor=3

	movers, "TG1" and 2nd movers, "TG2") (Johnson & Mislin, 2011, for a meta-analysis)	This amount is multiplied by a given factor and transferred to the 2nd movers.	(n=1)
8		A 2nd mover ("trustee") observes how much was transferred and decides how much of the transfer, if anything, to transfer back to the 2nd mover.	Observed transfers={1.6, 3.3, 5.1, 6.7, 8.3, 9.9}<br><br>(n=6, randomized order)
9	Money burning game ("MBG") (Zizzo & Oswald, 2001)	A pair of randomly matched counterparts decide how much of an endowment to spend to decrease the payoff of their counterparts. This amount is multiplied by a given factor and subtracted from the counterpart's payoff. Subjects earn nothing from "burning" their counterparts' payoff.	Endowment=€10.00<br><br>Multiplier factor={2,3,4,5} (order randomized)<br><br>(n=4, randomized order)
10	Entry game ("EG") (Camerer, 2003 for a review; Camerer	Randomly paired participants are to choose between the same two options: a safe but low paying option and a potentially higher paying but uncertain option. If both players choose the higher paying option, both earn 0. The only way for a player to earn the high paying option is if he/she chooses it and the counterpart does not. On the other hand, the low paying option results in a secure	Low paying option value varied between 0 and 15€, in steps of 1€<br><br>High paying option value was kept fixed at

	& Lovallo, 1999 for a connection between EGs and status)	payoff, regardless the choice of one's counterpart. Participants made several decisions but never received feedback on the outcome of any of them. The value of the high paying option was kept fixed, while the value of the low paying option varied for each decision.	15.00.<br><br>(n=16, randomized order)
11	Dilemma of the commons ("DOC") (Ostrom, Dietz, Dolsak, Stern, & Stonich, 2002, for a review)	Groups of three participants decide how much of take from a common resource. The amounts taken are then summed. If this sum exceeds a certain threshold, all players receive nothing. If the threshold is not exceeded, each player keeps what they took and the remaining sum in the common resource is first multiplied by a given factor and then split equally among the players, independently of what they took.	Common resource value=€30.00<br><br>Threshold=€18.00<br><br>Multiplier factor={2,3}<br><br>(n=2, no randomization)
12	Second party punishment ("2PP")	A first mover decides how much of an endowment to transfer to a randomly matched counterpart.<br><br>Participants play the role of the 2nd mover unaffected party, who observe how much is transferred and decide how much of an endowment to spend,	Endowment=€5.00<br><br>Multiplier factor=3<br><br>Observed transfers =

		if anything, to punish the 1st mover.<br><br>This amount is multiplied a given factor and subtracted from the 1st mover's payoff. Participants only played the 2PP as second movers.	{0.3, 1.5, 2.6, 3.5, 6.1, 7.5}<br><br>(n=6, randomized order)
13	Impunity game ("Imp") (Bolton & Zwick, 1995; Yamagishi et al., 2009)	The impunity game is identical to the ultimatum game with one exception: if recipients reject an offer, they earn nothing (as in the ultimatum game) however the proposer still keeps what he/she proposed. Subjects only played the impunity game as second movers.	Observed proposals={0.1, 0.9, 1.6, 2.9, 3.9 4.8}<br><br>(n=6, randomized order)
14	Charitable donations ("CD")<br><br>(Böckler, Tusche, & Singer, 2016; Hare, Camerer, Knoepfle, & Rangel, 2010)	Subject read short descriptions of several real life charitable organizations and decided how much of an endowment to donate. They were informed that a random decision would be selected and that they could keep 20% of what they did not donate.	Endowment=€50.00<br><br>(n=13, randomized order)
15	Zurich prosocial game ("ZPG")<br><br>(Böckler et al., 2016; Leiberg,	Two participants simultaneously navigate a computer maze with the objective of reaching a treasure worth a monetary prize. There are treasures, one for each participant, and there is no way for a player to obtain both. Occasionally, paths are blocked by	Prize=€0.5<br><br>(n=8, randomized order)

	Klimecki, & Singer, 2011)	locked doors and participants are to decide whether to you use one of their limited keys to open the door for their co-players or not.	
16*	Asymmetric dictator game ("ADG") (inspired from Dana, Cain, & Dawes, 2006)	The asymmetric dictator game is identical to the dictator game (see 1 in this table) with the exception that subjects (i.e., the "dictators") are told that recipients will never now any game ever took place. Instead, recipients are told that any additional money they receive is the result of a lottery.	Endowment=€10.00<br><br>(n=1)
17*	Compliance ("Comp") (inspired from (Klucharev, Hytönen, & Rijpkema, 2009)	Identical to the charitable donations (see 14 in this table) with the exception that participants are informed about the average donations of other participants, before making their choices.	Endowment=€50.00<br><br>(n=13, randomized order)

Table 1. Social economic games. For each game, we indicate, in parenthesis, which variable was derived: DG (amount transferred), CD (average donation size), T1 (amount entrusted), T2 (average amount returned), UG1 (amount proposed), UG2/Imp (percentage of rejections), EG/SH (percentage of choices to choose the uncertain option – i.e., "enter"/"cooperate"), MBG (percentage of decisions to "burn"), rDOC (average amount of taking, after reverse scaling, in order to favor comparability with the public good game), PG (average contribution), ZPG (percentage of decisions to help). For each decision, participants were told that they would be randomly re-matched to another participant in the room. Asterisks indicate two more exploratory games ("ADG" and "Comp") that we piloted here to be potentially be used in an upcoming "affiliation induction". As the two games in question were unrelated to this study's hypotheses, they were not further analyzed.

<table>
<tr><th></th><th>Game</th><th>Description</th><th>Game parameters
(number of trials)</th></tr>
<tr><td></td><td>Risk
(Bohnet & Zeckhauser, 2004)</td><td>Participants choose between two options, specifically two mixed gambles ("A" or "B"), each offering a 50-50 chance to gain/lose different monetary amounts. The two options were similar in expected value but differed in their "riskiness"/variance.</td><td>The gamble parameters were adapted from von Dawans et al. (2012) and were the following:
<table><tr><th>A</th><th>B</th></tr><tr><td>+5.2/+0.4,</td><td>+2.7/+2.3</td></tr><tr><td>+6.2/+0.4</td><td>+2.7/+2.3</td></tr><tr><td>+3.7/-1.1</td><td>+1.2/+0.8</td></tr><tr><td>+5.7/-1.1</td><td>+1.2/+0.8</td></tr><tr><td>+4.2/-0.4</td><td>+2.7/+2.3</td></tr><tr><td>+7.2/-0.4</td><td>+2.7/+2.3</td></tr><tr><td>+4.7/-1.1</td><td>+1.2/+0.8</td></tr><tr><td>+6.7/-1.1</td><td>+1.2/+0.8</td></tr></table>
(n=8, randomized order)</td></tr>
<tr><td></td><td>Loss aversion
(McCusker & Carnevale, 1995)</td><td>Participants are required to make a series of binary decisions, on whether to accept or reject a gamble. If participants accept the gamble they can either win or lose a given monetary amount, with equal probability.</td><td>The parameters were adapted from Gächter et al. (2007).

The gain magnitude was always kept fixed at €6.00.

Loss magnitude={-1,-2,-3,-4,-5,-6,-7}</td></tr>
</table>

<table>
<tr><td></td><td></td><td></td><td>(n=7, randomized order)</td></tr>
<tr><td></td><td>Temporal discounting (Stevens & Hauser, 2004)</td><td>Participants are to choose between a sooner but lower paying option and later but higher paying option.</td><td>Parameters were adapted from Rustichini et al. (2016).

The later-paying option value was kept fixed at 80€.

The sooner paying option value varied between 45€ and 75€, in steps of 5€.

There were 4 possible moments in which the sooner and later rewards could be delivered, these are listed in the table below:

<table><tr><th>Sooner</th><th>Later</th></tr><tr><td>now</td><td>tomorrow</td></tr><tr><td>now</td><td>in 1 week</td></tr><tr><td>in 1 week</td><td>in 1 week and 1 day</td></tr><tr><td>in 1 week</td><td>in 2 weeks</td></tr></table>
(n=28, randomized order)</td></tr>
</table>

Table 2. Non-social economic games. For each game, we indicate, in parenthesis, which variable was derived: risk (the percentage of risky choices), loss-aversion (the percentage of choices to gamble), temporal discounting (the percentage of patient decisions).

S3. Experimental demand

To control whether our results might have been affected by experimental demand, at the end of the experimental session, participants took part in an awareness questionnaire probing their possible insights into the objectives of the experiment.

Material. The questionnaire was inspired from Bargh & Chartrand's (2000) "funneled" questionnaire, in which participants are gradually made aware of elements in the experimental environment that might have affected their behavior. Specifically, participants provided open answers to the following 7 questions:

1. What is your next task?
2. What do you think are the goals this study?
3. Do you think one part of the study might have influenced another? If so, how and why?
4. Did you notice anything unusual about the experiment?
5. At any moment did you have the feeling of being deceived?

After the 5th question, participants were told that anticipated activities would actually not take place.

6. Did you already doubt that you would not [see the puppies/take part in the business simulation?]
7. Now that you know, what do you think the goal of this study was?

Methods. Two assistants independently read participants' open answers and, for each of the questions above, provided a rating, from 1 (no sign of suspicion) to 4 (relevant understanding), indicating how much they believed the participant had insight into the objective of the induction. After testing for inter-rater reliability and averaging over their ratings, we then re-ran our main analysis of interest using two exclusion criteria: a first excluded participants that understood how the announced activities were intended to affect their economic decisions (e.g., "The puppies made me nicer") (a score of 4), while a second more stringent criteria excluded participants that admitted that the activities could have affected their decisions, but not in a way that was pertinent to our experimental hypotheses (e.g., "the puppies made me happy") (a score of 3). If the participants provided no answer on a given question, or an irrelevant answer, raters were instructed to provide no rating.

This scoring system was used for all the questions above, except for question 1 ("what's the next task?"). For this question, a score of 1 indicated that the participant answered as expected (e.g., "going to the "puppy room", or taking part in the group activity), while a

score of 2 indicated that participants expressed doubts that the activity would actually take place. However, neither of the raters ever provided a score of 2 for this question, which we consequently disregarded for the remaining analysis. We thus focused on questions 2 through 5 (both included), that is, on any question that was asked before revealing that the announced activities would actually not take place.

Inter-rater reliability. We first addressed inter-rater reliability by running Pearson's correlations between the provided ratings, for each question separately. With one exception, the ratings positively and significantly correlated between the two raters (between r=0.28 and 0.71, all $p_s<0.05$). In fact, for the power induction only, the correlation between ratings was not significant for question 4 (which asked participants whether they noticed anything unusual during the experiment). However, upon examination of this item, it became evident that the non-significant correlation was due to very low variance on very low suspicion scores. Specifically, one rater provided only one "2" and otherwise provided scores of "1". Furthermore, neither of the raters ever provided a scores higher than 2 in question 4 of the power induction. Since this question did not enable to detect any suspicious participants, we discarded it from further analysis, and we averaged over the two ratings for each of the remaining questions.

Results. These suggested that 11 participants in the Care induction expressed relevant understanding on how the announced activities were intended to affect their behavior. We thus re-ran the cooperation and punishment models described in paper while excluding these participants. Both models retained the main effect of the induction (cooperation model: $F_{(2,180)}=5.88$, $p<0.01$; punishment model: $F_{(2,180)}=4.69$, $p<0.05$). Even when excluding participants that provided irrelevant explanations as to how the activities might have affected their decisions (which, together with the previously excluded ones amounted to N=25 in the Care induction and N=4 in the Power induction) both of the main effects of the inductions were retained (cooperation model: $F_{(2,162)}=5.73$, $p<0.01$; punishment model: $F_{(2,162)}=5.1$, $p<0.01$).

S4. Pseudo-randomization of the economic games

The economic games were distributed over two blocks and their order was pseudo-randomized. Specifically, there were three pairs of games that we believed were more likely to influence one another, as they were superficially similar to one another: 1) the 2nd player

and 3rd party punishment games, 2) the stag hunt and the entry game and 3) the dictator game and the asymmetric dictator game. In order to minimize potential spill-over between these superficially similar games, games within each of these pairs were always placed in distinct blocks (thus temporally farther away from one another). In addition to this, the charitable donations game was always placed at the end of the second block, as it adopted potentially compassion evoking charity descriptions that we suspected could affect subsequent choices. Finally, the Zurich Prosocial game was played after all of the economic games, as this was designed to investigate a more ecological form of decision making (Leiberg et al., 2011). The order of all the other economic games was fully randomized over the 2 blocks, for each participant, and the order of blocks was counter-balanced across participants.

S5. Motive and affect questionnaire

Self-reported motivational states and affect were measured before and after participants were told about the induction-related activities. Participants were provided with a list of words and for each one, they were asked to rate in how far they were "currently driven by these motivations, feelings or states", on a continuous visual analogue scale ranging -350 to 350 (numbers were not visible to participants). This was done with a novel questionnaire, which probed the motives of interest, that is, care and power, as well as five control motives (H. Heckhausen, 2008) or "emotivations" (Lerner, Li, & Valdesolo, 2015; Roseman, 2011), namely, achievement, affiliation, anger, fear and consumption. "Consumption" is a more exploratory motive, which we hypothesized could constitute a psychological parallel (e.g., Lea & Webley, 2006) to the "utility maximizing" motivation typically adopted in standard economics.

The Care-related items were "caring", "protective", "kind-hearted", "cordial", "helpful", "affectionate", "sympathetic", and "consoling"; the while power-related words were "mighty", "dominant", "authoritarian", "firm", "influential", "condescending" and "officious". The items for the remaining five motives were the following: anger ("aggressive", "angry", "offended", "irritable", "argumentative", "tempestuous", "spirited"), fear ("apprehensive", "afraid", "timid", "nervous", "panic-stricken", "overcautious", "frightened", "reserved"), achievement ("hard-working", "industrious", "capable", "efficient", "ambitious", "success-driven", "obstinate", "productive"), affiliation ("entertaining", "ingratiating", "excluded", "conventional", "attached", "obliging",

"popular") and consumption ("consumerist", "hoarding", "avaricious", "greedy", "materialistic", "cheap", "pleasure-seeking", "acquisitive", "desire to buy", "gluttonous"). These words were selected to represent each motive because they were found to be maximally discriminative of those motives (relative to each of the others) in a semantic categorization task described elsewhere (Chierchia, Parianen Lesemann, Snower and Singer, under revision). In addition to these motive-related constructs, affect was measured by asking participants to provide ratings on a number of items related either to happiness ("content", "happy", "overjoyed", "pleasant", "enthusiastic") or sadness ("sad", "downcast"). Overall, the questionnaire consisted of 63 items, to be aggregated into 7-motive related and 2 mood related measures. The order of the items was fully randomized for each participant, who viewed 7 items per page. To analyze this data, first, we subtracted pre-induction ratings from post-induction ratings, in order to obtain a "change score" for each item. Then, we averaged over the items related to each of the seven motives and the two moods, thus obtaining 9 difference change scores. Finally to assess motivational state changes and these difference scores were compared between inductions.

S6 Induced Power and gender

As illustrated in the description of the "punishment model" in the main manuscript, the interaction between gender and the induction was not significant in the omnibus test ($p=0.12$). However, when setting the reference level of the "induction" term (i.e., Care, Power or Control) to the Care (or Power) induction, the interaction estimate between gender and the Power induction (or Care) was marginally significant ($p=0.051$), which led us to probe this interaction further. Indeed, selected contrasts within the model suggested that Care and Power differentially affected punishments in males ($M=0.66$, 95% CI [0.14 1.19], $p<0.01$), not in females ($M=0.06$, 95% CI [0.43 0.56], $p=0.96$) (supplementary figure S1, panel A).

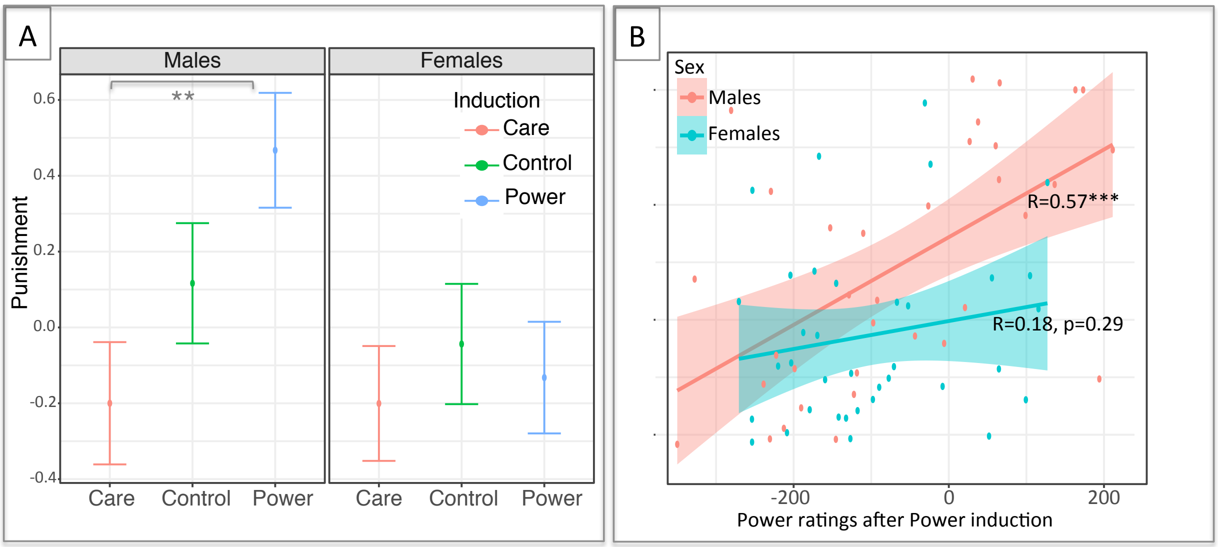

Supplementary figure S1. A. Induced Power and Care differentially affect punishments in males, not females. Model estimates predicting latent measures of punishment in economic decisions as a function of contextually induced Care or Power motives (vs. Control induction) and sex. Error bars represent 95% confidence intervals. **=p<0.01, *=p<0.05. **B. Self-reported feelings of power (after the Power induction) predict punishment in males not females.** ***=p<0.001.

To investigate this issue further, we ran another model on participants in the Power group only. We refer to this as a "Power model". This model investigated whether subjective feelings of power, as measured by the power ratings after the Power induction, predicted punishments in males but not females. Moreover, as the Power induction increased ratings of power but also of fear, we used this occasion to investigate whether feelings of fear might have also mediated the effects of the Power induction on punishments. Since power and fear ratings after the Power induction did not correlate (correlation coefficient=0.06, p=0.6), there was no multi-collinearity issue in using both of these terms as predictors in the same model. In synthesis, the Power model predicted punishment rates based on post-induction power and fear ratings (both standardized), and their interaction with gender.

This model revealed a significant main effect of gender ($F_{(1,64)}$=5.00, p<0.05), with males punishing at higher rates than females in the Power induction (M=0.5, 95% CI [0.05 0.93], p<0.05). It also revealed a highly significant impact of self-reported feelings of power on punishments ($F_{(1,64)}$=15.02, p<0.001). This latter effect was further qualified by a marginally significant interaction with gender ($F_{(1,64)}$=2.81, p<0.1). These findings were complemented

by simple (Pearson's) correlations between power ratings after the Power induction and punishments. In fact, while feelings of power predicted higher punishments in males (correlation coefficient=0.56, $t_{(32)}$=3.9, 95% CI [0.28 0.76], p<0.001), they did not in females (correlation coefficient=0.18, $t_{(34)}$=1.07, 95% CI [-0.16 0.48], p=0.2) (see Supplementary figure S1, panel B). Fear-ratings after the Power induction had no significant impact on punishments (p=0.73) and did not interact with gender (p=0.68), we thus disregard fear from further analysis and discussion. We briefly discuss these findings below.

We found that induced Power and Care differentially affected punishments in males but not females and that, similarly, self-reported feelings of power predicted punishments in males but not females. These results should be taken with a grain of salt, as we had not hypothesized them and the interaction between the induction and gender was not significant in the omnibus tests. However, they could provide preliminary insights into the relation between motives and gender. In fact, these results seem in line with research suggesting that males exhibit a relative preference for competitive environments (Croson, 2009; Marianne, 2011, for reviews) and that they are more sensitive to inter-group competition (Van Vugt et al., 2006). More speculatively, these findings could also be related to testosterone (Kouri et al., 1995; Mazur & Booth, 1998; Josephs et al., 2011; Eisenegger, 2012), which has been found to increase economic punishments in males (Burnham, 2007; Dreher, Dunne, & Pazderska, 2016; Zak et al., 2009) but not females (Eisenegger et al. 2010). Future research could investigate whether, indeed, contextual power predisposes males but not females towards punishments, and whether hormonal markers of endogenous testosterone mediate this.

S7. Instructions of the economic games and questionnaires.

Table of Contents

Self-reported motivational states and mood 18

Economic Games: Welcome text...19

Dictator game 20

Stag hunt..20

Stag hunt sample questions..21

Ultimatum game (first mover) 23
Ultimatum game (second mover) 23
Money burning game 24
Trust game 24
Trust sample questions 25
Computer trust 27
Public good game 27
GPG sample questions 28
Entry Game 30
Entry game sample questions 30
Second party punishment game 32
2pp sample questions 33
Third party punishment 34
Impunity game 34
Asymmetric Dictator 35
Common resource dilemma 35
Risk 36
Loss aversion 37
Temporal Discouting 37
Awareness questionnaire 38

Self-reported motivational states and mood

Inwiefern fühlen Sie sich in diesem Moment angetrieben durch die folgenden Motivationen und Gefühle? Bitte antworten Sie ganz spontan Ihrem Bauchgefühl nach. Um zu starten, klicken Sie die linke Maustaste.

In how far do you feel driven by the following motivations and feelings? Please answer spontaneously according to your gut feeling. To start, press the left mouse button.

Economic Games: Welcome text

Willkommen! Vielen Dank, dass Sie heute an unserer Studie zu ökonomischen Entscheidungen teilnehmen. Sie werden für Ihre Teilnahme in Bar bezahlt. Für jede Entscheidung, die Sie treffen, wird Ihnen per Zufall ein anderer Versuchsteilnehmer im Raum als Gegenüber zugeteilt. Sie und ihr Gegenüber bleiben dabei komplett anonym. Sie werden eine Reihe von Entscheidungen treffen. Allerdings werden wir Sie bis zum Ende des Experiments nicht über das Resultat Ihrer Entscheidungen informieren. Am Ende des Experiments wird per Zufall eine Ihrer Entscheidungen ausgewählt. Für diese Entscheidung bekommen Sie von uns einen Bonus ausgezahlt - ZUSÄTZLICH zu den 7€, die Sie pro Stunde verdienen. Die Höhe des Bonus hängt dabei von Ihrer Entscheidung und der Ihres Gegenübers ab. Da Sie bis zum Ende nicht wissen, welche Ihrer Entscheidungen relevant für Ihre Bezahlung ist, raten wir Ihnen, JEDE Entscheidung mit gleich hoher Aufmerksamkeit zu bedenken. Vor den Entscheidungssituationen werden wir Ihnen ein paar Probefragen stellen - es sei denn, wir teilen Ihnen explizit mit, dass Sie - ohne Probefragen - direkt zu den Entscheidungen kommen. Diese Probefragen stellen wir, um sicher zu gehen, dass Sie - und ihr Gegenüber - die Regeln, die für die momentane Entscheidung relevant sind, auch richtig verstanden haben. Wichtig: diese 'Probefragen' haben KEINERLEI Einfluss auf Ihre Bezahlung. Wenn Sie bereit sind, klicken Sie die linke Maustaste um zu starten. Sollten Sie irgendwelche Fragen haben, jetzt oder im Verlauf des Experiments, wenden Sie sich bitte an die Experimentleitung.

Welcome! Thank you for participating in our study about economic decisions. You will be paid in cash for your participation. For each decision you make, another participant in this room will be assigned at random to be your game counterpart. You and your counterpart will thereby remain completely anonymous. You will make a number of decisions. However, you will not be informed about the outcome of your decisions until the end of the experiment. At the end of the experiment, one of your decisions will be drawn at random. For this decision, you will get paid a bonus -in addition to the 7 € you are earning per hour-, which will depend on your choice and the choice of your counterpart. As you will not get to know which of your decisions will be relevant to your payment until the end of the experiment, we advise you to pay equally high attention to ALL of the decisions you make. Prior to all decisions, we will ask you some trial questions – unless noted that you will get to the main decisions without trial questions. The trial questions are asked to ensure that you - and your counterpart - correctly understood the rules relevant to the next

decision. Important: The trial questions do NOT AT ALL influence your payment. If you are ready, click the left mouse button to start. If you have any question now or at any moment during the experiment: Please raise your hand, and the experimenter will assist you.

Dictator game

Sie bekommen 10€ und können davon einen Teil an einen zufällig gewählten Versuchsteilnehmer im Raum überweisen. Dabei können Sie die gesamte Summe behalten, oder aber einen Teil oder alles an Ihr Gegenüber überweisen. Unabhängig von Ihrer Entscheidung erhält Ihr Gegenüber die Summe, die Sie ihm überweisen - Sie behalten den Rest. Für diese Entscheidung gibt es keine Probefragen. Sollten Sie noch Fragen haben, wenden Sie sich bitte jetzt an die Versuchsleitung. Andernfalls klicken Sie bitte um weiter zu kommen.

You will receive 10€ and can transfer a part thereof to a participant in the room selected by chance. You can thereby keep all of the money, or transfer some or all of the money to your counterpart. Independent of you decision, your counterpart receives the amount that you transfer – you keep the rest. There will be no trial questions for this decision. If you have any questions, please get in touch with the experimenter now. Otherwise, please click to proceed.

Stag hunt

Im Folgenden müssen Sie und Ihr per Zufall ausgewählter Gegenüber sich zwischen zwei Optionen entscheiden (A oder B). Sie sehen beide die selben Optionen. Wählen Sie Option A, erhalten Sie 15€ - allerdings NUR, wenn Ihr Gegenüber AUCH Option A wählt. Andernfalls (wählt er Option B), erhalten Sie 0€. Wenn Sie Option B wählen, erhalten Sie den jeweiligen Betrag (zwischen 0 und 15€) SICHER, unabhängig davon, für welche Option Ihr Gegenüber sich entscheidet. Klicken Sie, um mit den Probenfragen weiter zu machen.

In the following, you and your perchancely selected counterpart have to choose between two options (A or B). You both see the same options. If you choose option A, you receive 15€ -but ONLY IF your counterpart ALSO choses option A. Otherwise (if he chooses option B) you receive 0€. If you choose option B, you will FOR SURE receive the respective amount of money (0 to 15€), independent of the choice of your counterpart. Click to proceed with the trial questions.

Stag hunt sample questions

Zur Erinnerung: Sie und Ihr Gegenüber entscheiden zwischen zwei Optionen (A oder B).

Wählen Sie Option A, erhalten Sie 15€ - NUR, wenn Ihr Gegenüber AUCH Option A wählt.
Wählen Sie Option A, und Gegenüber Option B, erhalten Sie 0€.

Wählen Sie Option B, erhalten Sie den jeweiligen Betrag (zwischen 0 und 15€) SICHER.
Unabhängig von der Entscheidung Ihres Gegenübers.

Welche Option wählen Sie?

15 €, nur wenn beide

5 €, sicher

As a reminder: You and your counterpart choose between two options (A or B). If you choose option A, you receive 15€ -but ONLY IF your counterpart ALSO choses option A. If you choose option A, and your counterpart option B, you receive 0€. If you choose option B, you will FOR SURE receive the respective amount of money (0 to 15€), independent of the choice of your counterpart. Which option do you want to choose? (Choice is: Option A, 15€, 'only if both')

Wenn Ihr Gegenüber Option A wählt, wieviel Geld erhält er dann?

a) 5€, unabhängig davon, was ich wähle

b) 15€, wenn ich A wähle

c) 5€, wenn ich B wähle

d) 15€, unabhängig davon, was ich wähle

If you counterpart chooses option A, how much money does he receive?

- ***a) 5€, independent of my choice***
- ***b) 15€, if I also choose A***
- ***c) 5€, if I choose B***

d) 15€, independent of my choice

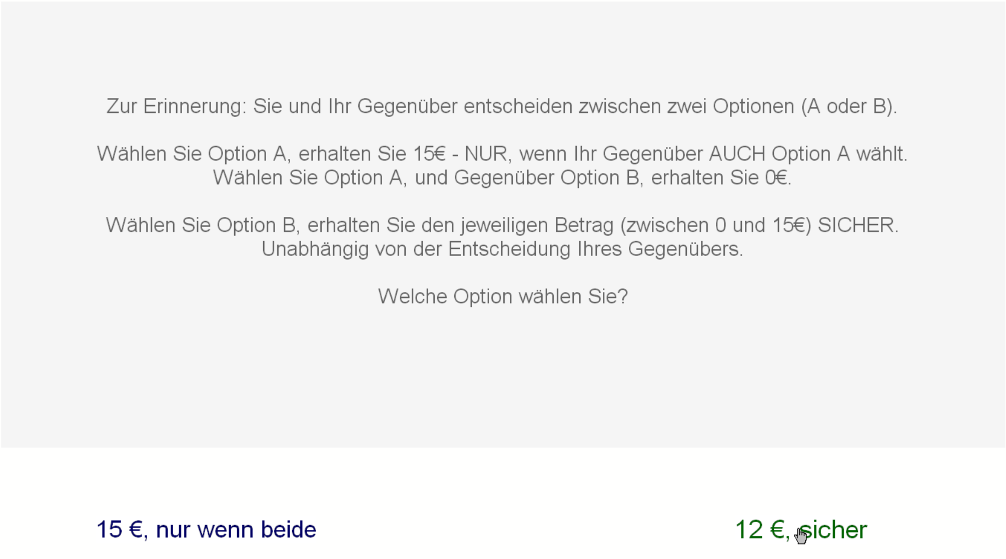

As a reminder: You and your counterpart choose between two options (A or B). If you choose option A, you receive 15€ -but ONLY IF your counterpart ALSO choses option A. If you choose option A, and your counterpart option B, you receive 0€. If you choose option B, you will FOR SURE receive the respective amount of money (0 to 15€), independent of the choice of your counterpart. Which option do you want to choose? (Choice is: Option B, 12€, 'for sure')

Wenn Sie oben Option B wählen, wieviel Geld erhalten Sie dann?

a) 11€, nur wenn mein Gegnüber auch B wählt

b) 15€, nur wenn mein Gegnüber A wählt

c) 11€, unabhängig davon, was mein Gegnüber wählt

d) 15€, wenn mein Gegnüber auch B wählt

If you choose option B above, how much money do you receive?

a) 11€, only if my counterpart also chooses B

b) 15€, only if my counterpart chooses A

c) ***11€, independent of my counterparts choice***
d) ***15€, if my counterpart also chooses B***

Ultimatum game (first mover)

Sie und Ihr per Zufall ausgewählter Gegenüber sind in folgender Situation: Eine Person ('Person A') erhält 10€, und kann 0 bis 10€ davon an eine anderen Person ('Person B') überweisen. Person B sieht wie viel Person A überweist und entscheidet das Angebot anzunehmen oder abzulehnen. Wenn Person B annimmt, wird der Betrag wie abgesprochen geteilt. Lehnt Person B ab, gehen beide leer aus (erhalten 0€). Für jede Entscheidungsrunde ist Ihnen ein anderer Versuchsteilnehmer im Raum zugeteilt.

You and your randomly selected counterpart are in the following situation: One Person (Person A) gets 10€ and can transfer a part of this sum (0€ to 10€) to the other person (Person B). Person B gets to know the sum transferred and accepts or rejects the offer. If Person B accepts, the sum will be shared as Person A proposed. If Person B rejects, both go empty handed (receive 0€). Prior to each round, another participant in this room will be randomly selected as your counterpart.

Ultimatum game (second mover)

Sie und Ihr per Zufall ausgewählter Gegenüber sind in folgender Situation: Eine Person ('Person A') erhält 10€, und kann 0 bis 10€ davon an eine anderen Person ('Person B') überweisen. Person B sieht wie viel Person A überweist und entscheidet das Angebot anzunehmen oder abzulehnen. Wenn Person B annimmt, wird der Betrag wie abgesprochen geteilt. Lehnt Person B ab, gehen beide leer aus (erhalten 0€). Für jede Entscheidungsrunde ist Ihnen ein anderer Versuchsteilnehmer im Raum zugeteilt.

You and your randomly selected counterpart are in the following situation: One Person (Person A) receives 10€ and can transfer a part of this sum (0 to 10€) to another Person (Person B). Person B will get to know how much money was transferred by A and will decide whether to accept or reject the offer. If B accepts, the two players will divide the money as proposed by player A. If B rejects, both players will receive 0€. In each round you play, you will be matched with a different randomly designated participant in this room.

Money burning game

In der folgenden Situation sind Sie einem zufällig gewählten Versuchsteilnehmer aus dem Raum zugeteilt. Sie beide lesen die exakt selben Instruktionen: Sie können bis zu 10€ dazu benutzen das Guthaben eines per Zufall ausgewählten Gegenübers aus dem Raum zu verringern. Jeder Euro den Sie einsetzen, reduziert das Guthaben Ihres Gegenübers um jeweils unterschiedliche Beträge. Zum Beispiel, wenn der 4-fache Betrag abgezogen wird, würden 2 € die Sie zahlen, das Guthaben Ihres Gegenübers um 8 € verringern - und wenn Sie in der gleichen Situation 3 € einsetzen, verringert sich sein Guthaben um 12 €, usw. Sie können dabei kein zusätzliches Geld verdienen. Alles was Sie nicht ausgeben, behalten Sie. Sollten Sie noch Fragen haben, wenden Sie sich jetzt bitte an die Versuchsleitung. Es gibt keine Probefragen: auf der nächsten Seite werden Sie und Ihr Gegenüber ihre Entscheidungen treffen.

In the following situation you will be matched with a randomly designated participant in this room. You and your counterpart will be reading the exact same instructions, which are the following: You can use up to 10€ to reduce the balance of a randomly selected counterpart in the room. Every euro you employ will be reducing the balance of your counterpart by different amounts of money. For instance, if the reduction in fourfold, 2€ spent will reduce your counterparts balance by 8€ - if you spend 3 € in the same situation, you will reduce your counterparts balance by 12€ etc. You can earn no additional money by reducing the balance of others. You will keep all money you have not spent. If you have any questions, please get in touch with the experimenter. There will be no practice questions: At the next page, you and your counterpart will take your decisions.

Trust game

Sie sind mit einer per Zufall gewählten Versuchsperson aus dem Raum in der folgenden Situation: Einer von Ihnen ('Person A') erhält 10€ und kann einen Teil davon an den Anderen ('Person B') überweisen. Person B erhält daraufhin die dreifache Summe des Betrags den Person A überwiesen hat. Dann kann Person B einen Teil an Person A zurücküberweisen. Um mit den Probefragen weiter zu machen, klicken Sie bitte.

You and a randomly selected counterpart are in the following situation: One of you (Person A) gets 10€ and can transfer any part of that to the other (Person B). Person B receives the threefold amount of money that Person A has transferred. Consequently,

Person B may transfer a part of the money back a part to Person A. Please click to proceed with the trial questions.

Trust sample questions

Sie sind mit einer per Zufall gewählten Versuchsperson aus dem Raum in der folgenden Situation:

Einer von Ihnen ('Person A') erhält 10€ und kann einen Teil davon
an den Anderen ('Person B') überweisen.

Person B erhält daraufhin die 3-fache Summe des Betrags den Person A überwiesen hat.

Dann kann Person B einen Teil an Person A zurücküberweisen.

You and a randomly selected counterpart are in the following situation: One of you (Person A) gets 10€ and can transfer any part of that to the other (Person B). Person B receives the threefold amount of money that Person A has transferred. Consequently, Person B may transfer a part of the money back a part to Person A.

Person A überweist 10 € an Person B. Wieviel könnte Person B jetzt zurück überweisen?

a) Alles zwischen 0€ und 15€

b) Alles zwischen 5€ und 10€

c) Alles zwischen 0€ und 30€

d) Alles zwischen 3€ und 30€

Person A transfers 10€ to Person B. How much money could Person B now transfer back?

a) Anything between 0€ and 15€

b) Anything between 5€ and 10€

c) **Anything between 0€ and 30€**

d) **Anything between 3€ and 10€**

Sie sind mit einer per Zufall gewählten Versuchsperson aus dem Raum in der folgenden Situation:

Einer von Ihnen ('Person A') erhält 10€ und kann einen Teil davon
an den Anderen ('Person B') überweisen.

Person B erhält daraufhin die 3-fache Summe des Betrags den Person A überwiesen hat.

Dann kann Person B einen Teil an Person A zurücküberweisen.

Person A überweist 5 Euro an Person B, und Person B überweist 7€ zurück. Wie viel Geld würden beide also am Ende erhalten?

a) Person A gewinnt 7€, Person B gewinnt 8€

b) Person A gewinnt 17€, Person B gewinnt 8€

c) Person A gewinnt 17 €, Person B gewinnt 13€

d) Person A gewinnt 12€, Person B gewinnt 13€

Person A transfers 5€ to Person B, and Person B transfers 7€ back to A. How much money would both receive in the end?

a) ***Person A gains 7€, Person B gains 8€***
b) ***Person A gains 17€, Person B gains 8€***
c) ***Person A gains 17€, Person B gains 13€***
d) ***Person A gains 12€, Person B gains 13€***

Computer trust

Für diese Entscheidung erhalten Sie 10€. Sie können jeglichen Betrag (0-10€) davon an einen Zufallsgenerator überweisen. Jeglicher Betrag, den Sie überweisen, wird daraufhin VERDREIFACHT. Danach wird der Generator Ihnen einen zufällig gewählten Anteil der resultierenden Summe zurücküberweisen. Jegliche Summe die Sie nicht überweisen, behalten Sie. Es gibt keine Probefragen für diese Entscheidung. Um DIREKT zu den Bonus-relevanten Entscheidungen zu gelangen, klicken Sie bitte.

For this decision you will receive 10€. You can transfer any part of this sum (0-10€) to a random number generator. The amount you transfer will be tripled. Afterwards, the number generator will transfer back to you a randomly selected part of the resulting sum. You can keep whatever you have not transferred. There will be no trial questions for this decision. Please click, to DIRECTLY proceed with the bonus-relevant decisions.

Public good game

Für diese Entscheidung sind Ihnen 3 weitere Versuchspersonen per Zufall zugeordnet. Sie alle lesen die selben Instruktionen: Jeder von Ihnen erhält 30€ und kann davon jeglichen Betrag in einen Gruppenfond einzuzahlen. Sie können also alles, einen Teil oder nichts von Ihren 30€ einzahlen. Die Summe, die Sie nicht einzahlen, behalten Sie. Nachdem alle 4 Teilnehmer Ihre Entscheidung getroffen haben, wird der resultierende Gesamtbertrag im Fond verdoppelt oder -dreifacht, und danach gleichmäßig auf alle Teilnehmer aufgeteilt und ausgezahlt, Dies gilt UNABHÄNGIG davon, wieviel jeder vorher eingezahlt hat. Sollten Sie noch Fragen haben, wenden Sie sich bitte an die Versuchsleitung - andernfalls klicken Sie, um zu den Probefragen zu gelangen.

For this interaction you will be randomly matched with 3 other participants in the room. You are all reading these same instructions: Each of you will receive 30€ and may deposit any part of this sum into a (group-) stock. You can transfer a part, nothing or all of your 30€. You will keep everything you did not transfer. After all 4 participants have made their decisions, the resulting sum in the stock will be multiplied by an indicated number. The final sum will then be equally divided among the 4 people and disbursed, INDEPENDENTLY of how much everyone had contributed. If you have any questions, please contact the experimenter. Else click to proceed with the trial questions.

GPG sample questions

Zur Erinnerung: Sie und 3 weitere Versuchspersonen haben 30€ und
können 0-30€ davon in einen Gruppenfond einzahlen.

Der Betrag in dem Fond wird VER-2-FACHT und gleichmäßig
an alle ausgezahlt - unabhängig davon, wieviel jeder eingezahlt hat.

Wieviel Geld von 30 € möchten sie in den Fond einzahlen ?

As a reminder: You and three other participants received 30€ and may deposit any part of this into a (group-) stock. The resulting sum in the stock will be multiplied by 2 and disbursed to all participants in equal parts - INDEPENDENTLY of how much everyone had contributed. How much of 30€ do you want to deposit into the stock?

Wieviel Geld enthält der Fond in dieser Runde, wenn alle Spieler 5 € beitragen.

a) 40€

b) 60€

c) 20€

d) 5€

How much money does the stock in this round if all players deposit 5€.

a) 40€

b) 60€

c) 20€

d) 5 €

Zur Erinnerung: Sie und 3 weitere Versuchspersonen haben 30€ und
können 0-30€ davon in einen Gruppenfond einzahlen.

Der Betrag in dem Fond wird VER-3-FACHT und gleichmäßig
an alle ausgezahlt - unabhängig davon, wieviel jeder eingezahlt hat.

Wieviel Geld von 30 € möchten sie in den Fond einzahlen ?

As a reminder: You and three other participants received 30€ and may deposit any part of this into a (group-) stock. The resulting sum in the stock will be multiplied by 3 and disbursed to all participants in equal parts - INDEPENDENTLY of how much everyone had contributed. How much of 30€ do you want to deposit into the stock?

Wieviel Geld enthält der Fond in dieser neuen Runde, wenn alle Spieler 10 € beitragen.

a) 40€

b) 60€

c) 100€

d) 120€

How much money does the stock in this round if all players deposit 10€.

a) 40€

b) 60€

c) 100€

d) 120€

Entry Game

Im Folgenden müssen Sie und Ihr zufällig ausgewählter Gegenüber sich zwischen zwei Optionen entscheiden (A oder B). Sie sehen beide die selben Optionen. Wählen NUR Sie Option A, erhalten Sie 15€. Wählen Sie BEIDE Option A, erhalten beide 0€. Wählen Sie Option B, erhalten Sie in jedem Fall den angegebenen Wert (zwischen 0 und 15€) - unabhängig davon, für welche Option sich Ihr Gegenüber entscheidet. Klicken Sie, um zu den Probefragen zu kommen.

In the following, you and your randomly selected counterpart have to choose between two options (A or B). You both see the same options. If ONLY YOU are choosing option A, you will receive 15€. If BOTH of you are choosing option A, you will both receive 0€. If you chose option B, you will FOR SURE receive the amount indicated (0 to 15€), independent of your counterparts choice. Click to proceed with the trial questions.

Entry game sample questions

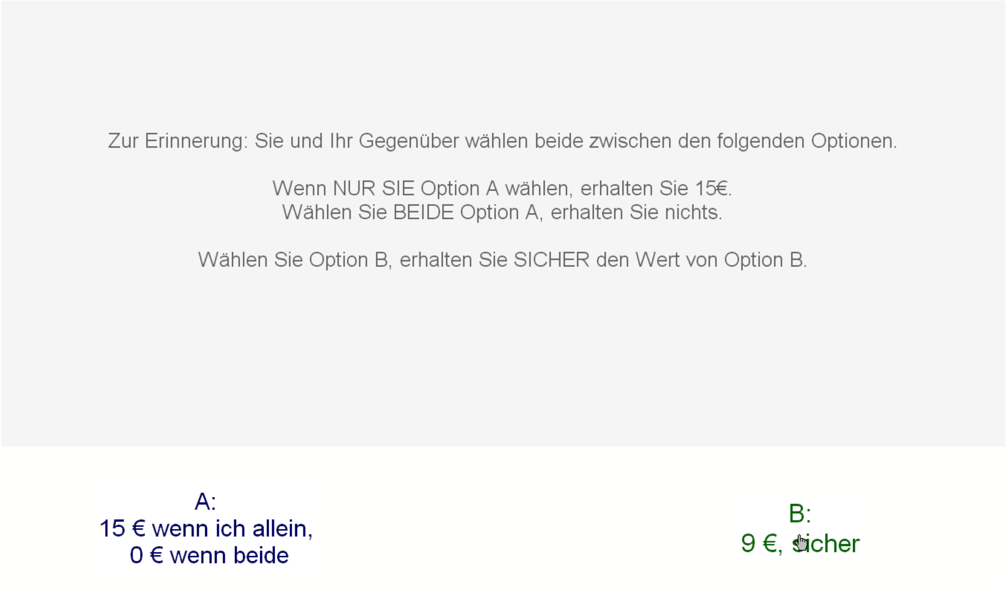

As a reminder: You and your counterpart are both choosing between the following option. If ONLY YOU are choosing option A, you will receive 15€. If BOTH of you are choosing

option A, you will receive 0€. If you chose option B, you will FOR SURE receive the value of option B.

Wenn Sie oben Option B wählen, wieviel Geld erhalten Sie dann?

a) 6€, nur wenn der andere Spieler B wählt

b) 15€, nur wenn der andere Spieler A wählt

c) 6€, unabhängig davon, was Spieler B wählt

d) 15€, wenn der andere Spieler B wählt

If you, in the example above, choose option B, how much money do you receive?

- ***a) 6€, only if the other person chooses B***
- ***b) 15€, only if the other person chooses A***
- ***c) 6€, independent of the other persons choice***
- ***d) 15€, if the other person chooses B***

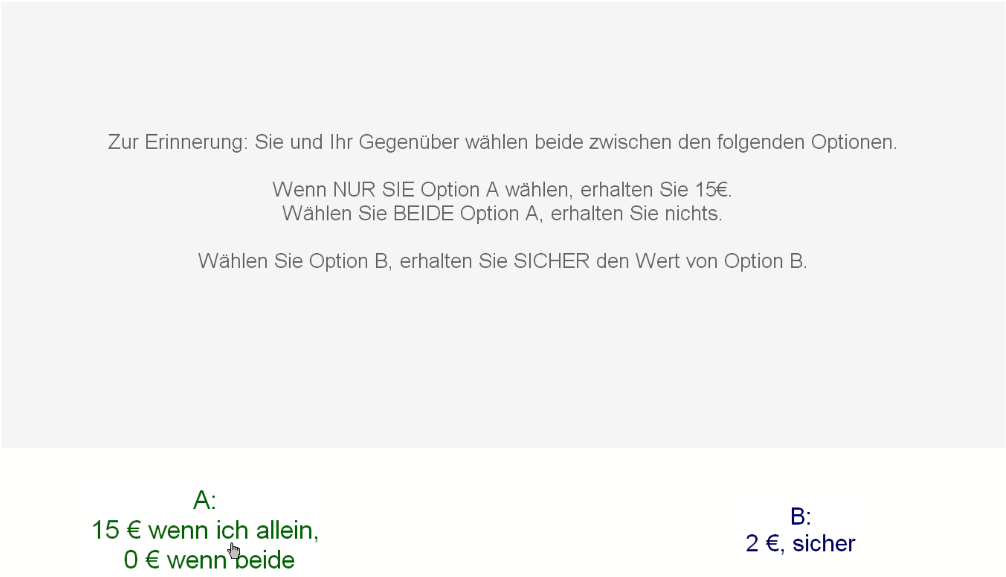

As a reminder: You and your counterpart are both choosing between the following option. If ONLY YOU are choosing option A, you will receive 15€. If BOTH of you are choosing option A, you will receive 0€. If you chose option B, you will FOR SURE receive the value of option B.

Wenn Ihr Gegenüber Option A wählt, wieviel Geld erhält er dann?

a) 0€, wenn ich B wähle

b) 15€, unabhängig davon, was ich wähle

c) 15€, wenn ich auch A wähle

d) 0€, wenn ich auch A wähle

If your counterpart chooses option A, how much money does he receive?

a) ***0€, if I choose B***
b) ***15€, independent of what I choose***
c) ***15€, if I also choose A***
d) ***0€, if I also choose A***

Second party punishment game

Sie wurden per Zufall einem Gegenüber aus dem Raum zugeordnet. Ihr Gegenüber erhält jetzt 15€ - Sie bekommen 5€.Ihr Gegenüber wird dann entscheiden, ob er oder sie Ihnen einen Teil der 15€ überweist. Was Ihr Gegenüber Ihnen nicht überweist, behält er oder sie selbst. Nachdem Sie erfahren haben, wie viel Ihr Gegenüber Ihnen überwiesen hat, haben Sie die Möglichkeit, jeglichen Teil Ihrer 5€ dazu einzusetzen, um das Guthaben Ihres Gegenübers zu verringern. Jeder Euro, den Sie einsetzen, reduziert das Guthaben von Ihrem Gegenüber um je 3€. Sie können dabei kein Geld gewinnen. Alles Geld, was Sie nicht einsetzen, behalten Sie. Klicken Sie, um zu den Probefragen zu gelangen.

You have been matched with a randomly designated counterpart. Your counterpart receives 15€ - you receive 5€. Your counterpart will then decide whether he/she transfers a part of the 15€ to you. He/she will keep whatever was not transferred. After you got to know how much your counterpart transferred to you, you will have the opportunity to use any part of your 5€ to reduce the balance of your counterpart. Every euro you employ herein reduces the balance of your counterpart by 3€. You cannot earn money by reducing the balance of your counterpart. You keep all money that you don't employ in reducing the balance of your counterpart. Click to proceed with the trial questions.

2pp sample questions

Ein per Zufall ausgewählter Gegenüber aus dem Raum hat 15€ erhalten,
die er/sie nun mit Ihnen teilen kann.

Danach haben Sie die Möglichkeit, bis zu 5€ einzusetzen,
um das Guthaben ihres Gegenübers zu veringern.

1 Euro den Sie einsetzen reduziert das Guthaben von Ihrem Gegenüber um je 3€.

Klicken Sie, um zu den Probefragen zu gelangen.

A randomly designated counterpart has received 15 € which he/she may share with you. Afterwards,you will have the opportunity to use any part of your 5€ to reduce the balance of your counterpart. Every euro you employ herein reduces the balance of your counterpart by 3€. Click to proceed with the trial questions.

Wenn Sie 3€ ausgeben, damit Ihrem Gegenüber etwas von seinem Guthaben abgezogen wird, um wieviel reduziert sich sein Guthaben dann?

a) 6€

b) Das hängt davon ab, wieviel er mir überwiesen hat

c) 9€

d) gar nichts

If you spent 3€ to reduce the balance of your counterpart, by what amount of money will his balance be reduced?

a) 6€
b) depends on how much he has transferred to me
c) 9€

d) nothing

Um wieviel Geld können Sie das Guthaben Ihres Gegenübers insgesamt vermindern?

a) 3€

b) 15€

c) 5€

d) Das hängt davon ab, wieviel er mir überwiesen hat

What is the highest amount of money by which you can reduce your counterparts balance?

a) 3€
b) 15€
c) 5€
d) Depends on how much he has transferred to me

Third party punishment

Sie beobachten folgende Entscheidungssituation zwischen zwei zufällig gewählten Versuchsteilnehmern im Raum: Person A erhält 15 Euro, und kann davon 0 bis 15€ an Person B überweisen. Daraufhin sehen Sie, wie viel Person A and Person B überwiesen hat, und können dann bis zu 5 Euro dafür verwenden, das Guthaben von Person A zu reduzieren. Jeder Euro, den Sie einsetzen, verringert das Guthaben von Person A um 3€.

You are observing the following situation between two participants in the room selected by chance: Person A receives 15€ and can transfer 0 to 15€ to Person B. You will get to know how much money Person A transferred to Person B and you will then have the possibility to use up to 5€ to reduce the balance of Person A. Every Euro that you employ will reduce the balance of Person A by 3€.

Impunity game

Ein per Zufall ausgewählter Gegenüber aus dem Raum hat 10€ erhalten. Er oder sie kann Ihnen davon nun jeglichen Teil überweisen. Nachdem Sie sehen, wie viel Ihr Gegenüber Ihnen überwiesen hat, können Sie das Angebot annehmen oder ablehnen. Sollten Sie ablehnen, erhalten Sie 0€ und Ihr Gegenüber bekommt noch immer den Betrag, der ihm laut seines Teilangebots zusteht. Klicken Sie, um direkt zu den Entscheidungen zu gelangen.

A randomly selected counterpart in the room received 10€. He or she can now transfer any part of the sum to you. You will get to know how much your counterpart transferred to you and you may then accept or reject the offer. If you reject, you will receive nothing and your counterpart will still receive the amount he allotted to himself. Click here to directly proceed with your decisions.

Asymmetric Dictator

Sie bekommen 10€ und können davon einen Teil an einen zufällig gewählten Versuchsteilnehmer im Raum überweisen. Ihnen steht dabei frei, ob Sie alles, einen Teil, oder nichts von Ihrem Geld überweisen. Ihr Gegenüber erhält die Summe, die Sie ihm überweisen - Sie behalten den Rest. WICHTIG: nur Sie lesen diese Instruktionen. Ihr Gegenüber hingegen, liest in seinen Instruktionen, dass er an einer Lotterie teilnimmt, die mit gleich hoher Wahrscheinlichkeit einen Betrag zwischen 0 und 10 € auswählt. In Wirklichkeit bestimmt aber Ihre Entscheidung, wie viel Geld Ihr Gegenüber erhält. Sollten Sie noch Fragen haben, wenden Sie sich bitte jetzt an die Versuchsleitung. Andernfalls klicken Sie, um DIREKT zu den Entscheidungen zu gelangen, die für Ihren Bonus relevant sein könnten (es gibt keine Probefragen!).

You receive 10€ and you can decide how much of this sum you want to transfer to a randomly selected counterpart in the room. You may transfer a part, nothing or all of the 10€. Your counterpart receives what you transfer to him – you will keep the rest. IMPORTANT: Note that only you are reading these instructions. Your counterpart instead reads that he participates in a lottery which will randomly determine a monetary gain between 0 and 10. As a matter of fact, however, you are the one who determines the earnings of your counterpart. If you have any questions, please ask the experimenter. Otherwise please click to directly proceed with the bonus-relevant decisions (there are no trial questions!).

Common resource dilemma

Für diese Entscheidung sind Ihnen drei weitere, zufällig gewählte Versuchspersonen aus dem Raum zugeteilt. Sie alle lesen die selben Instruktionen: Es gibt einen gemeinsamen Gruppenfond in dem 30€ liegen. Sie müssen nun entscheiden, ob Sie einen Teil des Geldes aus dem Fond nehmen. Sie können dabei jeglichen Betrag zwischen 0 und 12€ aus dem Fond nehmen. ABER: Sollten Sie und Ihre Gegenüber zusammen mehr als 18€ aus dem Fond nehmen, gehen alle leer aus. Im Gegenzug: Sollte der Betrag, den Sie gemeinsam abgehoben

haben, 18€ NICHT überschreiten, kann jeder den herausgenommenen Betrag behalten. Zusätzlich dazu wird der im Fond verbleidende Betrag VERZWEI / DREI/ VIER-FACHT und gleichmäßig an alle 4 Teilnehmer ausgezahlt. Dies gilt UNABHÄNGIG davon, wieviel jeder vorher herausgenommen hat. Es gibt für diese Entscheidung keine Probefragen. Bei Unklarheiten melden Sie sich bitte bei der Experimentleitung. Andernfalls: Wieviel Geld von 30€ möchten Sie aus dem Fond nehmen?

For this decision, you are matched with 3 randomly selected participants in the room. You are all reading the same instructions: There is a common stock with contains 30€. You have to decide whether you take a part of the money out of this stock. You may take any amount of money between 0€ and 12€. BUT: If you and your 3 counterparts take in sum MORE THAN 18€, then everyone will leave empty-handed. On the other hand, if the sum taken from the stock does not exceed 18€, all participants may keep the money they took. Additionally, the remaining sum in the stock gets multiplied by 2(/3/4) and equally paid out to all 4 participants. This is independent of how much each participant had contributed. There will be no trial questions for this decision. If you have any questions, please ask the experimenter. Otherwise: How much out of 30€ do you want to take out of the stock?

Risk

In der folgenden Situation ist Ihnen KEIN Gegenüber zugeordnet. Sie müssen sich zwischen zwei Optionen entscheiden (A oder B). Jede Option beinhaltet dabei eine Lotterie mit zwei möglichen Ausgängen. Nachdem Sie sich für eine Lotterie entschieden haben, wird per Zufall (50%) ein Ausgang ausgewählt. Zum Beispiel: Lotterie A: 5,70€ / -1,10€ Lotterie B: 1,20€ / 0,80€ Entscheiden Sie sich hier für Lotterie A, gewinnen Sie 5,70€ oder verlieren 1,10€; Entscheiden Sie sich hingegen für die Lotterie B, gewinnen Sie 1,20€ oder 0,80€. Sollten Sie noch Fragen haben, melden Sie sich beim Versuchsleiter. Ansonsten klicken Sie bitte, um DIREKT zu den Entscheidungen zu gelangen, die für Ihren Bonus relevant sein könnten (es gibt keine Probefragen!).

For the following decision, you are NOT matched with a counterpart. You will have to choose between two options (A or B). Each option consists of a lottery with 2 possible outcomes. The outcome will be randomly chosen (50 %) after you have taken your decision. For example: Lottery A: 5.70€/-1.10€ Lottery B: 1.20€/0.80€. If you choose Lottery A, you win 5.70€ or lose 1.10€; if you choose Lottery B, you win 1.20€ or 0.80€. If

you have any questions, please get in touch with the experimenter. Otherwise please click to directly proceed with the bonus-relevant decisions (there are no trial questions!).

Loss aversion

Für die folgende Entscheidungssituation wird Ihnen KEIN Gegenüber zugeordnet .Ihr Gewinn hängt also nur von Ihren Entscheidungen ab - sowie von dem 'Wurf einer Münze' (50/50% Chance). Sie starten in diese Entscheidungsrunde mit einem Guthaben von 10€, und haben die Möglichkeit, mit diesem Geld an einer Lotterie teilzunehmen. Abhängig von Ihren Entscheidungen und dem Resultat der 50/50-Lotterie, besitzen Sie danach mehr oder weniger Geld, als die anfänglichen 10€. Zum Beispiel: Entscheiden Sie sich, an der Lotterie 'Gewinn = 6, Verlust = 3' teilzunehmen, werden Sie entweder (50% Chance) 3€ verlieren, oder (ebenso 50% Chance) 6€ gewinnen, und am Ende in diesem Fall 7€ oder 16€ besitzen. WICHTIG: Ihr Gewinn oder Verlust sammelt sich NICHT über mehrere Runden, da am Ende des Experiments nur EINE Entscheidung per Zufall für Ihren Bonus ausgewählt wird. Sie starten also in jede Lotterie wieder mit einem Guthaben von 10€. An welcher der folgenden Lotterien möchten Sie teilnehmen? (Klicken Sie, um zu den Entscheidungen zu gelangen)

For the following decision, you will NOT be matched with a counterpart. Your payoff will thus depend on your choice only, and on the result of a coin flip (50/50 % chance). At the beginning of the round, your balance will be 10€, and you will have the opportunity spend this money in a lottery. Depending on your decisions and the outcome of the lottery, you might end up with more or less money than the 10€ you started out with. The chance of winning the lottery is 50 % in all rounds. For example: If you decide to take part in the lottery 'Gain=6, Loss=3', you will either lose 3€ (with a 50 % chance) or gain 6€ (with a 50% chance). In the end, your balance will be either 7€ or 16€. IMPORTANT: Your losses and earnings are not accumulated over all rounds because only ONE round will be selected for payment at the end of the experiment. You thus start with a balance of 10€ at the beginning of each round of the lottery. In which of the following lotteries do you want to participate? (Click here to get to the decisions).

Temporal Discouting

In diesem Spiel können Sie entweder heute eine bestimmte Summe Geld erhalten, oder später eine größere Summe. Für welche der beiden Optionen entscheiden Sie sich?

In this game, you can either now receive a specific amount of money or later get a sum even larger. For which of the two options are you going?

Awareness questionnaire

Jetzt noch ein paar kurze Fragen zu Ihrem Hintergrund – bitte geben Sie Ihre Antwort jeweils mittels der Tastatur ein und bestätigen dann mit 'Enter'. Um zu starten, klicken Sie bitte die Maustaste.

Now a few questions about your background - please enter your response by means of the keypad and confirm with 'Enter'. Please click the mouse to start.

Wie alt sind Sie?

How old are you?

Was ist Ihr Geschlecht? (m/w)

What is your gender?

Wie viele Jahre sind Sie zur Schule gegangen? Und wie viele Jahre haben Sie in einer weiterführenden Ausbildung/im Studium verbracht? (Beispiel: 10+3)

How many years have you been to school? And how many years have you taken part in continuing education/how many years have you studied? (Example: 10+3)

Und nun ein paar allgemeine Fragen zum heutigen Studienverlauf. Bitte geben Sie Ihre Antwort weiterhin mittels der Tastatur ein (freier Text, gerne kurz/in Stichworten) und bestätigen dann mit 'Enter'.

And now a few general questions about todays study. Please keep entering your answer by means of the keypad (free text, gladly short/in bulletpoints) and confirm with 'Enter'.

1. Was ist Ihre nächste Aufgabe?

1. What is your next task?

2. Was denken Sie, sind die Ziele der Studien, an denen Sie heute teilnehmen?

2. What do you think are the goals of the studies in which you are participating today?

3. Meinen Sie, dass ein Teil der Studien an denen Sie heute teilgenommen haben, einen anderen Teil beeinflusst haben könnte? Wenn ja, wie und warum?

3. Do you think some part of todays study might have influenced another? If yes, how and why?

4. Ist Ihnen an den Studien irgendetwas als ungewöhnlich aufgefallen?

4. Did you notice anything unusual about the experiments?

5. Hatten Sie an irgendeinem Moment das Gefühl, getäuscht zu werden?

5. Did you have the feeling of being deceived at any moment during the study?

A n diesem Punkt der Studie können wir Ihnen mitteilen, dass das Feedback, dass Sie erhalten haben, nicht auf Ihren Essay bezogen war. Stattdessen haben alle Versuchsteilnehmer die gleiche Rückmeldung bekommen. Statt eines Rückmeldegesprächs wird die Studie deshalb hier enden. Bitte bewahren Sie darüber noch Stillschweigen und sprechen Sie es nicht laut aus, da die anderen Versuchsteilnehmer von diesem Ablauf erst später erfahren. Die Versuchsleitung wird Sie auch gleich noch dazu auffordern, 'dann bitte jetzt noch zum letzten Teil der Studie mitzukommen' . Dies ist, um die anderen Versuchsteilnehmer nicht zu verwirren. Jetzt wo Sie wissen, dass der letzte Teil der Studie 2 wegfällt:

At this point of the study, we can tell you that the feedback you received was not related to your essay. Instead, all participants got the exact same feedback. Therefore, the last part of study 2 will not take part and the experiment will end now. Please keep this information to yourself and do not talk about it loudly, as the other participants in the will only get to know this later on. Also, the experimenter will still ask you to 'please come and join me for the last part of the study' We thereby want to prevent confusion of the other participants. Now that you know that the last part of study 2 won't take place:

6. Hatten Sie schon vorher Zweifel daran, dass die Studie so abläuft wie angekündigt?

6. Did you already doubt that the study would take place as announced, earlier?

7. Und jetzt wo Sie wissen, dass die beiden Studien an denen Sie teilgenommen haben, hiermit abgeschlossen sind: Was meinen Sie, waren die Ziele der heutigen Studien?

7. And now that you know that the two studies you participated in are over after this questionnaire: what do you think were the goals of today's studies?

References

Bargh, J., & Chartrand, T. (2000). The mind in the middle. In *Handbook of research methods in social and personality psychology* (pp. 253–285).

Böckler, A., Tusche, A., & Singer, T. (2016). The Structure of Human Prosociality Differentiating Altruistically Motivated, Norm Motivated, Strategically Motivated, and Self-Reported Prosocial Behavior. *Social Psychological and Personality Science*, *7*(6), 530–541.

Bohnet, I., & Zeckhauser, R. (2004). Trust, risk and betrayal. *Journal of Economic Behavior & Organization*.

Bolton, G., & Zwick, R. (1995). Anonymity versus punishment in ultimatum bargaining. *Games and Economic Behavior*.

Burnham, T. C. (2007). High-testosterone men reject low ultimatum game offers. *Proceedings.Biological Sciences / The Royal Society*, *274*(1623), 2327–2330.

Camerer, C. F. (2003). Behavioural studies of strategic thinking in games. *Trends in Cognitive Sciences*, *7*(5), 225–231.

Camerer, C., & Lovallo, D. (1999). Overconfidence and Excess Entry: An Experimental Approach. *American Economic Review*, *89*(1), 306–318. http://doi.org/10.1257/aer.89.1.306

Dana, J., Cain, D. M., & Dawes, R. M. (2006). What you don't know won't hurt me: Costly (but quiet) exit in dictator games. *Organizational Behavior and Human Decision Processes*, *100*(2), 193–201.

Dreher, J., Dunne, S., & Pazderska, A. (2016). Testosterone causes both prosocial and antisocial status-enhancing behaviors in human males. *Proceedings of the*.

Eisenegger, C., Naef, M., Snozzi, R., Heinrichs, M., & Fehr, E. (2010). Prejudice and truth about the effect of testosterone on human bargaining behaviour. *Nature*, *463*(7279), 356–359.

Engel, C. (2010). Dictator Games: A Meta Study. *SSRN Electronic Journal*. http://doi.org/10.2139/ssrn.1568732

Fehr, E., & Fischbacher, U. (2004). Third-party punishment and social norms. *Evolution and Human Behavior*, *25*(2), 63–87.

Gächter, S., Johnson, E., & Herrmann, A. (2007). Individual-level loss aversion in riskless and risky choices.

Hare, T. a, Camerer, C. F., Knoepfle, D. T., & Rangel, A. (2010). Value computations in ventral medial prefrontal cortex during charitable decision making incorporate input

from regions involved in social cognition. *The Journal of Neuroscience : The Official Journal of the Society for Neuroscience*, *30*(2), 583–90. http://doi.org/10.1523/JNEUROSCI.4089-09.2010

Heckhausen, H. (2008). *Motivation and action*. (J. Heckhausen & H. Heckhausen, Eds.) (Second). New York, NY, US: Cambridge University Press.

Johnson, N., & Mislin, A. (2011). Trust games: A meta-analysis. *Journal of Economic Psychology*.

Klucharev, V., Hytönen, K., & Rijpkema, M. (2009). Reinforcement learning signal predicts social conformity. *Neuron*, *61*(1), 140–151.

Lammers, J., Galinsky, A. D., Gordijn, E. H., & Otten, S. (2011). Power Increases Social Distance. *Social Psychological and Personality Science*. http://doi.org/10.1177/1948550611418679

Lea, S. E. G., & Webley, P. (2006). Money as tool, money as drug: The biological psychology of a strong incentive. *Behavioral and Brain Sciences*, *29*(2), 161–209.

Leiberg, S., Klimecki, O., & Singer, T. (2011). Short-term compassion training increases prosocial behavior in a newly developed prosocial game. *PLoS One*, *6*(3), e17798.

Lerner, J., Li, Y., & Valdesolo, P. (2015). Emotion and decision making. *Annual Review of Psychology*, *66*, 799–823.

McCusker, C., & Carnevale, P. (1995). Framing in resource dilemmas: Loss aversion and the moderating effects of sanctions. *Organizational Behavior and Human Decision*.

Ostrom, E., Dietz, T., Dolsak, N., Stern, P., & Stonich, S. (2002). *The drama of the commons*.

Roseman, I. J. (2011). Emotional Behaviors, Emotivational Goals, Emotion Strategies: Multiple Levels of Organization Integrate Variable and Consistent Responses. *Emotion Review*, *3*(4), 434–443. http://doi.org/10.1177/1754073911410744

Stevens, J., & Hauser, M. (2004). Why be nice? Psychological constraints on the evolution of cooperation. *Trends in Cognitive Sciences*.

Yamagishi, T., Horita, Y., Takagishi, H., Shinada, M., Tanida, S., & Cook, K. S. (2009). The private rejection of unfair offers and emotional commitment. *Proceedings of the National Academy of Sciences of the United States of America*, *106*(28), 11520–11523.

Zak, P. J., Kurzban, R., Ahmadi, S., Swerdloff, R. S., Park, J., Efremidze, L., … Matzner, W. (2009). Testosterone administration decreases generosity in the ultimatum game. *PLoS One*, *4*(12), e8330.

Zelmer, J. (2003). Linear public goods experiments: A meta-analysis. *Experimental Economics*.

Zizzo, D. J., & Oswald, A. J. (2001). Are people willing to pay to reduce others' incomes? *Annales d'Economie et de Statistique*, 39–65.
